# Environmental selection, rather than neutral processes, best explain regional patterns of diversity in a tropical rainforest fish

**DOI:** 10.1038/s41437-023-00612-x

**Published:** 2023-03-30

**Authors:** Katie Gates, Jonathan Sandoval-Castillo, Chris J. Brauer, Peter J. Unmack, Martin Laporte, Louis Bernatchez, Luciano B. Beheregaray

**Affiliations:** 1 Molecular Ecology Laboratory, College of Science and Engineering, Flinders University, Adelaide, SA 5042 Australia; 2 Centre for Applied Water Science, Institute for Applied Ecology, University of Canberra, Canberra, ACT 2601 Australia; 3 Institut de Biologie Intégrative et des Systèmes (IBIS), Université Laval, 1030 avenue de la Médecine, Québec, Québec, G1V 0A6 Canada; 4 Ministère des Forêts, de la Faune et des Parc (MFFP) du Québec, Québec, QC Canada

**Keywords:** Evolution, Genetics

## Abstract

To conserve the high functional and genetic variation in hotspots such as tropical rainforests, it is essential to understand the forces driving and maintaining biodiversity. We asked to what extent environmental gradients and terrain structure affect morphological and genomic variation across the wet tropical distribution of an Australian rainbowfish, *Melanotaenia splendida splendida*. We used an integrative riverscape genomics and morphometrics framework to assess the influence of these factors on both putative adaptive and non-adaptive spatial divergence. We found that neutral genetic population structure was largely explainable by restricted gene flow among drainages. However, environmental associations revealed that ecological variables had a similar power to explain overall genetic variation, and greater power to explain body shape variation, than the included neutral covariables. Hydrological and thermal variables were the strongest environmental predictors and were correlated with traits previously linked to heritable habitat-associated dimorphism in rainbowfishes. In addition, climate-associated genetic variation was significantly associated with morphology, supporting heritability of shape variation. These results support the inference of evolved functional differences among localities, and the importance of hydroclimate in early stages of diversification. We expect that substantial evolutionary responses will be required in tropical rainforest endemics to mitigate local fitness losses due to changing climates.

## Introduction

Empirical studies are fundamental to the advancement of evolutionary theory and are increasingly relevant as we grapple with the novel selective forces of anthropogenic environmental change. Both adaptive and non-adaptive processes contribute to the proliferation of biodiversity, but there remains much to explore about their relative roles (Bernatchez 2016; Wellenreuther and Hansson 2016; Luikart et al. 2018). At a landscape scale, the environment is expected to modulate interactions between evolutionary mechanisms, namely natural selection, genetic drift, and gene flow (Haldane 1948; Slatkin 1987; Manel et al. 2003; Storfer et al. 2007). However, we are only now developing frameworks to untangle coexisting signatures of these processes in natural populations. Such studies are particularly sparse in biodiversity hotspots such as tropical rainforests, where there has not only been substantial debate about diversifying processes (Endler 1982; Mayr and O’Hara 1986; Moritz et al. 2000) but also suggestions of high risk to adaptive diversity from human influences (Moritz 2002; Barlow et al. 2018; França et al. 2020).

As some of the world’s most biodiverse and temporally continuous ecosystems, tropical environments merit a central place in eco-evolutionary research. Tropical rainforests alone may contain more than half the world’s species (Turner 2001; Primack and Corlett 2005) and are among the greatest terrestrial providers of ecosystem services (Brandon 2014). Attributes such as localised endemism, high niche specificity and history of relative stability may increase threats to diversity under environmental change (Reed 1992; Barlow et al. 2018; Hoffmann et al. 2019). However, there is an inherent logistical difficulty in studying such diverse and often remote ecological communities (Beheregaray 2008; Beheregaray et al. 2015; Clarke et al. 2017), and both terrestrial and freshwater tropics remain remarkably understudied relative to temperate ecosystems (Beheregaray et al. 2015; Wilson et al. 2016). There has also been a long history of contention about the processes generating and sustaining tropical rainforest biodiversity (Endler 1982; Mayr and O’Hara 1986; Haffer 1997; Smith et al. 1997). Biogeographic and palaeoecological research has debated factors permitting both the accumulation of species and the preconditions for divergence; while strong evidence suggests that the stability of rainforest refugia through glacial maxima has helped sustain high species richness (Weir and Schluter 2007; Weber et al. 2014; Cattin et al. 2016), the factors precipitating diversification remain less clear. Arguments for vicariant influences such as refugial isolation and landscape breaks (Wallace 1854; Haffer 1969; Vuilleumier 1971; Mayr and O’Hara 1986; Ayres and Clutton-Brock 1992; Dias et al. 2013) have been increasingly contested with evidence for parapatric and sympatric divergence across ecotones (Endler 1982; Smith et al. 1997; Kirschel et al. 2011; Cooke et al. 2012a, Cooke et al. 2012b, Cooke et al. 2014; Morgan et al. 2020).

While providing important geographical context, polarisation of such views presented in earlier research has sometimes obscured the complexity of evolutionary processes in rainforest taxa (Butlin et al. 2008; Jardim de Queiroz et al. 2017). For example, the difficulty of inferring adaptation in isolated populations against a neutral ‘null hypothesis’ may have encouraged the view that allopatric divergences were largely drift-driven, despite evidence that local selection can often be more effective in a low gene flow context (Schluter 2001; Nosil 2012; Beheregaray et al. 2015). Moreover, while species-level diversification has received great emphasis, intraspecific approaches are comparatively underexploited for identifying evolutionary processes such as drift and adaptation (Moritz et al. 2000; Moritz 2002). In tropical studies explicitly assessing neutral and adaptive processes, both have been found important for generating genetic or physiological variation (Freedman et al. 2010; Smith et al. 2011; Cooke et al. 2014; Brousseau et al. 2015; Benham and Witt 2016; Maestri et al. 2016; Termignoni‐García et al. 2017; Zhen et al. 2017; Gallego‐García et al. 2019; Morgan et al. 2020; Hay et al. 2022). This highlights the need for more nuanced assessments of rainforest diversity, which can be aided by increased integration of molecular and geospatial methods (Moritz et al. 2000; Moritz 2002; Beheregaray et al. 2015).

The field of landscape genomics has exploited rapidly advancing genomic and geospatial toolsets to detect ecological adaptation (Manel and Holderegger 2013; Hoffmann et al. 2015; Li et al. 2017), including in aquatic ecosystems (Grummer et al. 2019). Genotype-environment association (GEA) analyses have proven to be a powerful means to identify candidate loci under selection by specific environmental factors (Rellstab et al. 2015; Waldvogel et al. 2020), even for relatively weak allele frequency shifts (Bourret et al. 2014; Laporte et al. 2016; Forester et al. 2018). Similarly, phenotype-environment associations (PEAs) can allow the identification of ecologically adaptive phenotypes, benefited by multivariate approaches like geometric morphometrics (Zelditch et al. 2012; Maestri et al. 2016). Detection of local adaptation is complicated by the expectation of additional random, and potentially neutral, divergences, so statistical methods correcting for shared population history can benefit these approaches (Gautier 2015; Rellstab et al. 2015). For PEAs, it is also important to consider that plastic responses to environment, rather than evolved differences, can produce divergent physical characteristics (Merilä and Hendry 2014). Therefore, clearer interpretations can be made where it is possible to relate ecologically adaptive genotypes to significant phenotypic polymorphisms (Hu et al. 2020). Such integrative genotype-phenotype-environment (GxPxE) associations increase the opportunity for teasing apart eco-evolutionary mechanisms, and, by closing the gap between genotype, phenotype, and environment, can strengthen inferences about candidate genes underlying ecological adaptations (Smith et al. 2020; Carvalho et al. 2021).

Landscape heterogeneity places unique constraints on the biodiversity structure of taxa with restricted niches, including freshwater obligates. In contrast to temperate ecosystems, high year-round precipitation in tropical rainforests makes freshwater habitats ubiquitous, and their biotic interactions inextricable from those of the broader forest (Lo et al. 2020). However, available habitats and opportunities for gene flow in freshwater are typically restricted to dendritic, hierarchical, island-like, or ephemeral water features (Lévêque 1997; Grummer et al. 2019). The architecture of river networks and the strength and direction of flows can profoundly influence evolutionary dynamics (Paz‐Vinas et al. 2015; Thomaz et al. 2016; Brauer et al. 2018), as well as vulnerability to fragmentation (Jiménez-Cisneros et al. 2014; Davis et al. 2018; Brauer and Beheregaray 2020). These factors make understanding the spatial distribution of aquatic diversity important but complicated, and few riverscape genomic studies have been attempted in tropical freshwater (but see Barreto et al. 2020; Gallego‐García et al. 2019).

We therefore capitalise on growing knowledge of eco-evolutionary processes in Australian rainbowfishes (*Melanotaenia* spp, family Melanotaeniidae; e.g. McGuigan et al. 2003; McGuigan et al. 2005; Smith et al. 2013; McCairns et al. 2016; Gates et al. 2017; Brauer et al. 2018; Lisney et al. 2020; Sandoval-Castillo et al. 2020; Smith et al. 2020; Brauer et al. 2023). In this genus, previous work has indicated not only the likely importance of hydroclimate as a driver of diversity, but the utility of integrative methods for assessing aquatic adaptation. Early work found heritable and potentially convergent body shape variation in association with streamflow (*M. duboulayi*, *M. eachamensis*; McGuigan et al. 2003; McGuigan et al. 2005). More recently, experimental assessments of gene expression have detected selection for plasticity of thermal response mechanisms (*M. duboulayi*, *M. fluviatilis*, and *M. s. tatei*) (Smith et al. 2013; McCairns et al. 2016; Sandoval-Castillo et al. 2020). Riverscape GEAs have also supported intraspecies ecological divergence related to hydroclimate for *M. splendida tatei* (Attard et al. 2022), for hybridising *Melanotaenia spp*. across an elevational gradient (Brauer et al. 2023), for *M. fluviatilis* (Brauer et al. 2018) and *M. duboulayi* (Smith et al. 2020), with the latter including evidence of GxPxE links.

**Fig. 1:**
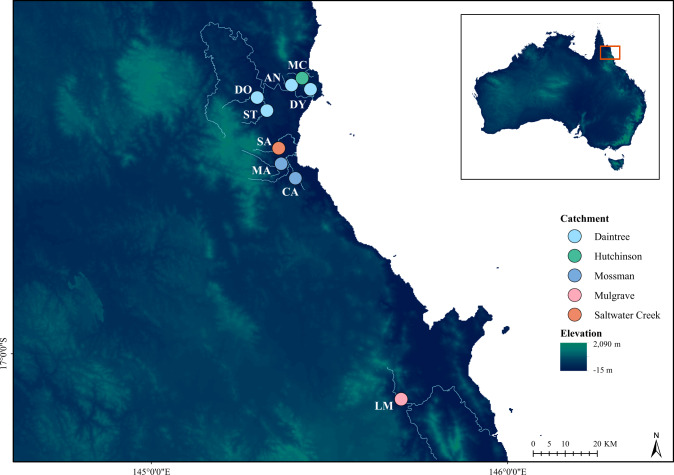
Sampling location map of *Melanotaenia splendida splendida* collected from the Wet Tropics of Queensland. Point colours correspond to river drainage of origin. Blue lines highlight only the sampled creeks and major rivers of each represented drainage system. Inset: extent indicator of main map relative to the Australian continent. Locality codes: LM Little Mulgrave Creek, CA Cassowary Creek, MA Marrs Creek, SA Saltwater Creek, ST Stewart Creek, DO Douglas Creek, DY Doyle Creek, AN Forest Creek, MC McClean Creek.

Despite these advances, genome-wide research has not yet been presented for a tropical representative of the clade. Hence, we focus this study on *Melanotaenia splendida splendida* (eastern rainbowfish), endemic to tropical north-eastern Australia. The species is abundant throughout its distribution, including several river systems in the complex rainforest landscape of the Wet Tropics of Queensland World Heritage Area (Pusey et al. 1995; Russell et al. 2003; Hilbert 2008; see Fig. 1 for sampled rivers). It inhabits a variety of freshwater environments, and is also known for its high morphological diversity, even within connected drainages (Pusey et al. 2004). Although the ecological relevance of this diversity has not yet been tested, the low to moderate dispersal tendency of *Melanotaenia* spp (Brauer et al. 2018; Smith et al. 2020) makes localised adaptation a plausible contributor. Moreover, the rugged terrain of the Great Dividing Range provides diverse conditions and possible selective influences across the sampled habitat (Nott 2005; Pearson et al. 2015). In that region, temperature, precipitation, and streamflow vary with latitude, elevation, terrain structure, and proximity to the coast (Metcalfe and Ford 2009; Stein et al., 2011), and human impacts according to land use (Pert et al. 2010). This environmental and climatic heterogeneity, combined with the recognised biodiversity values, make the Wet Tropics of Queensland an ideal location for testing hypotheses about evolutionary dynamics in tropical freshwaters.

The broad aims of this study were to develop an understanding of adaptive and neutral influences on variation in tropical rainforest freshwater ecosystems. This was approached using landscape genomics to characterise spatial patterns of genetic and morphological diversity, identify links between genotype, phenotype, and environment, and test the impacts of adaptive and non-adaptive forces on divergence across a variable rainforest hydroclimate. Based on previous evidence for climatic factors promoting adaptive diversity among higher latitude rainbowfishes (Brauer et al. 2018; Sandoval-Castillo et al. 2020; Smith et al. 2020), we tested the hypothesis that hydroclimate would also play a strong role in driving intra-species diversity within a tropical ecotype. The following questions were addressed: First, to what extent are genetic and morphological diversity correlated with hydroclimate beyond expectations based on neutral genetic structure? Second, if ecological associations exist, can further associations be drawn to suggest a genetic (heritable) adaptive component to the relevant morphology? Third, to what extent does catchment structure in this rugged terrain contribute to patterns of divergence? These factors have implications not only for understanding contemporary evolutionary processes in rainforest ecosystems, but also for the interpretation of adaptive resilience to environmental change.

## Methods

### Sample collection

During March 2017, wild *Melanotaenia splendida splendida* (eastern rainbowfish) were sampled from nine rainforest creek sites across five drainages in the Wet Tropics of Queensland, north-eastern Australia (Fig. 1; Supplementary Table S1a). Sites were chosen to maximise sampling coverage across the climatic habitat gradient, while targeting accessible creeks with known occurrences of *M. s. splendida*. Live fish were captured by seine netting and transported by road in closed containers fitted with battery-running air pumps to a mobile fieldwork station. Here, 267 fish were euthanised, one at a time, via an overdose of anaesthetic sedative (AQUI-S^®^: 175 mg/L, 20 min). Of these, 208 individuals (avg. ~23, min. 19 per sampling site; Table S1a) were photographed immediately after death for morphometric data collection (details in Supplementary Methods S1a). Fin clips from all 267 individuals were preserved in 99% ethanol and stored at −80°, of which 210 high quality samples were selected for the final DNA dataset (avg. ~23, min. 20 per site; Table S1a). For 180 individuals (avg. ~20, min. 15 per site), both genomic and morphometric datasets were of high quality, allowing direct comparisons in later GxPxE analyses.

### DNA extraction, library preparation and sequencing

We extracted DNA from fin clips using a salting-out protocol modified from Sunnucks and Hales (1996) (S1b). DNA was assessed for quality using a NanoDrop spectrophotometer (Thermo Scientific), for integrity using gel electrophoresis (agarose, 2%), and for quantity using a Qubit fluorometer (Life Technologies). High-quality samples from 212 individuals were used to produce double-digest restriction site-associated DNA (ddRAD) libraries in-house following Peterson et al. (2012) with modifications according to Sandoval‐Castillo et al. (2018) (S1c), which have demonstrated efficacy for rainbowfishes (e.g., Brauer et al. 2018). Samples were randomly assigned across sequencing lanes with an average of six replicates per lane for quality control. Four lanes were sequenced at the South Australian Health and Medical Research Institute Genomics Facility on an Illumina HiSeq25000 (single-ended), and one lane at Novogene Hong Kong on an Illumina HiSeq4000 (paired-ended).

### Bioinformatics: read trimming, alignment to genome, variant calling, and filtering

We used TRIMMOMATIC 0.39 as part of the DDOCENT 2.2.19 pipeline (Puritz et al. 2014) to demultiplex and trim adaptors from raw sequences, as well as leading and trailing low-quality bases (Phred < 20). Individuals with < 700,000 reads were considered poorly sequenced and were removed from the dataset. Sequences were mapped to a reference genome of the closely related *M. duboulayi* (Beheregaray et al. unpublished data; Brauer et al. 2023) following the GATK 3.7 pipeline (Van der Auwera and D O’Connor 2020). Briefly, we used BOWTIE 2.3.4 (Langmead and Salzberg 2012) to generate a FASTA file reference index and sequence dictionary from the genome and align individual sequences to the reference. After sorting and converting SAM files to BAM format, potential mapping errors and alignment inconsistencies were corrected using a local realignment around indels. Finally, variants were called from the mapped reads using BCFTOOLS 1.9 (Li 2011). To target high-quality SNPs, we used VCFTOOLS 0.1.15 (Danecek et al. 2011) to filter poorly sequenced reads, non-biologically informative artefacts (*sensu* O’Leary et al. (2018), variants other than SNPs (e.g., indels), and sites with a high likelihood of linkage disequilibrium (full details S1d).

### Differentiating putatively neutral versus outlier loci

Conformity of loci to neutral expectations was assessed using BAYESCAN 2.1 (Foll and Gaggiotti 2008), which identifies outlier loci under selection based on allele frequency distributions. Because the model relies on *F*_ST_, it requires prior specification of population membership. We therefore ran an analysis using FASTSTRUCTURE 1.0 (Raj et al. 2014) for the full filtered dataset (details S1e). We then ran _BAYESCAN_ using default settings for all filtered loci, with individuals assigned to putative populations based on the best *K* selected by FASTSTRUCTURE. A putatively neutral dataset was inferred using a false discovery rate <0.05. Such an approach is usually considered appropriate for minimally biased assessments of demographic parameters (Luikart et al. 2003; Luikart et al. 2018). The resulting dataset (14,478 loci, 210 individuals) was used for subsequent analyses of neutral genetic diversity and population structure except where otherwise specified.

### Genetic diversity and inference of population structure

We estimated neutral genomic diversity for each sampling site using ARLEQUIN 3.5 (Excoffier and Lischer 2010), including mean expected heterozygosity (*H*_*e*_), mean nucleotide diversity (π), and proportion of polymorphic loci (*PP*). We also calculated Wright’s fixation indices (*F*-statistics) in R (RC Team 2019) using HIERFSTAT 0.04–22 (Goudet 2005) for the entire sampling region. The same package was used to calculate pairwise *F*_ST_ and site-specific *F*_ST_ among sampling localities. To produce an overview of phylogenetic relationships among individuals, a Neighbour-Joining tree was constructed in PAUP* 4.0 (Swofford and Sullivan 2003) using TN93 distances (Tamura and Nei 1993). We also produced a scaled covariance matrix of sampling site-based ‘population’ allele frequencies (Ω) using BAYPASS 2.2 (Gautier 2015) core model, based on the full SNP dataset rather than the neutral subset. We further interrogated population structure using clustering approaches, including FASTSTRUCTURE, and Discriminant Analysis of Principal Components (DAPC) in R package ADEGENET 2.0.0 (Jombart 2008; Jombart and Ahmed 2011). Finally, we estimated asymmetrical rates of recent migration (m) between inferred populations using BA3-SNPS 1.1 (Wilson and Rannala 2003; Mussmann et al. 2019), a Bayesian Markov chain Monte Carlo approach. Full details of the above analyses, including preparation of input files, are in Supplemental Methods (S1f).

### Characterising environmental variation

Environmental variables characterising the regional aquatic landscape (resolution of 9 s/250 m) were obtained from the National Environmental Stream Attributes v1.1.3, a supplementary product of the Australian Hydrological Geospatial Fabric (Stein et al. 2011). From >400 available attributes, we selected only those which varied among sampling sites, were uncorrelated, were measured at a relevant scale, and were considered to have broad ecological relevance for freshwater organisms (further details S1g). The six selected variables were: stream segment aspect (ASPECT), river disturbance index (RDI), average summer mean runoff (RUNSUMMERMEAN), average annual mean rainfall (STRANNRAIN), average annual mean temperature (STRANNTEMP), and total length of upstream segments calculated for the segment pour-point (STRDENSITY) (Table S1g, Fig. S1g). These were used as a basis for the subsequent analyses of genotype-environment associations (GEA), phenotype environment associations (PEA) and GxPxE associations.

### Genotype-environment associations

We used GEAs to assess the effect of environment on genotype of *M. s. splendida* within the climatically heterogeneous Daintree rainforest. Since GEA approaches can vary markedly in their detection of candidate genes depending on demography, sampling design, and strength of selection (de Villemereuil et al. 2014; Rellstab et al. 2015; Forester et al. 2018), we chose to use two analytical approaches with different advantages. These included a Bayesian hierarchical model (BAYPASS 2.2 auxiliary covariate model; Gautier 2015), and constrained ordination (redundancy analysis; RDA) performed in R package VEGAN 2.5–6 (Oksanen et al. 2019). For both methods, we tested associations between the full SNP dataset (14,540) and the six scaled, uncorrelated environmental variables (see above) while controlling for putatively neutral genetic variation. The algorithm used by BAYPASS is well suited to study systems involving nested or hierarchical population structure (Gautier 2015), which is particularly common in dendritic habitats such as freshwater (Thomaz et al. 2016). We tested for GEA associations accounting for assumed population demographic structure (scaled sampling site-based allelic covariance; Ω), previously identified using the software’s core model (details in Supplemental methods S1h). Meanwhile, RDAs have been shown to have both a low rate of false positives and a high rate of true positives under a range of demographic histories, sampling designs, and selection intensities when compared with other popular GEA methods (Forester et al. 2018). Despite these strengths, we do emphasise that all detected environmental associations remain putative due to the unknown and potentially confounding factors present in natural study systems.

We first ran a global RDA using the full SNP dataset as the multivariate response matrix, and the six environmental variables (Fig. S1g), centred and scaled, as the explanatory matrix. Then, to control for demographic structure, partial RDAs (pRDAs) were used to model relationships between alternative (neutral) explanatory variables and genotypic responses, ordinating only the residual genotypic responses against environmental explanatory variables. To this end, three pRDAs were performed to include different neutral (or neutral proxy) covariable matrices, (1) significant principal components (PCos) of scaled Ω, (2) significant PCos of pairwise *F*_ST_, and (3) significant PCos of waterway distances. For each pRDA, we used the full set of SNP genotypes as a response matrix, and an explanatory matrix containing only environmental variables previously associated with genotype (*p* < 0.1) in the global RDA (full details S1h).

### Geometric morphometric characterisation and analyses

**Fig. 2:**
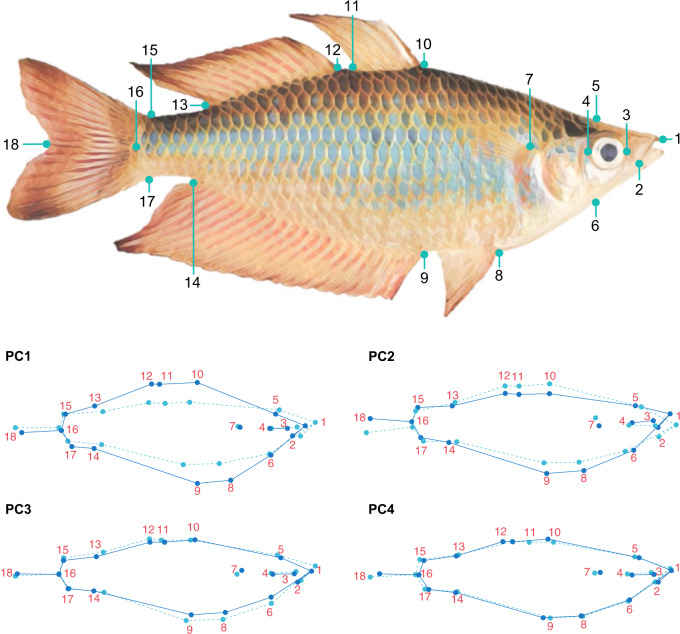
UPPER: The 18 landmarks used for geometric morphometric analysis of the eastern rainbowfish *Melanotaenia splendida splendida*. 1: Anterior tip of head, where premaxillary bones articulate at midline; 2: Posterior tip of maxilla; 3: Anterior margin in maximum eye width; 4: Posterior margin in maximum eye width; 5: Dorsal margin of head at beginning of scales; 6: Ventral margin at the end of the head; 7: Dorsal insertion of pectoral fin; 8: Anterior insertion of the pelvic fin; 9: Anterior insertion of the anal fin; 10: Anterior insertion of the first dorsal fin; 11: Posterior insertion of the first dorsal fin; 12: Anterior insertion of the second dorsal fin; 13: Posterior insertion of the second dorsal fin; 14: Posterior insertion of the anal fin; 15: Dorsal insertion of the caudal fin; 16: Posterior margin of the caudal peduncle (at tip of lateral line); 17: Ventral insertion of the caudal fin; 18: Posterior margin of the caudal fin between dorsal and ventral lobes. LOWER: Fig. 4. Wireframe graphical representation of significant principal components of body shape variation based on 18 landmarks for 207 *Melanotaenia splendida splendida* individuals sampled across nine rainforest sampling localities in the Wet Tropics of Queensland. Dark and light blue frames respectively represent body shapes at high and low extremes of each significant axis (scale factor = 0.75).

Eighteen landmarks were positioned on digital images of *M. s. splendida* collected during field sampling using TPSDIG2 2.31 (Rohlf, 2017). Landmarks (Fig. 2) were selected to maximise anatomical homology, repeatability, and representation of potentially ecologically relevant characteristics, based on recommendations by Zelditch et al. (2012) and Farré et al. (2016) (details S1i). Digitised TPS files were imported into MORPHOJ 1.07a (Klingenberg 2011) for exploratory analyses. Individual landmark configurations were subjected to Procrustes superimposition, that is, a scaling of homologous coordinates by size, rotation, and placement in space. The dataset was checked for outliers to ensure correct order and location of landmarks, and a covariance matrix was generated for the full dataset of individual Procrustes fits. To characterise major features of shape variation, a PCA was performed on the resulting covariance matrix. Due to size variation among individuals, an allometric regression was used to test the association between size (log centroid) and shape (Procrustes coordinates), pooled within population-based subgroups earlier identified by neutral genetic analyses. While allometric shape differences can result from adaptive evolution, ontogenic allometry may also be observed throughout the lifespan (Pélabon et al. 2013), potentially confounding signals of selection. Therefore, due to a strong relationship between size and shape (Supplementary Fig. S1i), residuals from size regression were used for the subsequent canonical variate analyses (CVAs), also performed in MORPHOJ. To test for relationships between body shape and locality of origin, we ran CVAs of Procrustes distances against sampling site and against catchment. This method calculates the total variation among groups, scaling for relative within-group variation. Statistical significance was assessed using 1000 permutation rounds.

### Phenotype-environment associations

To assess the effect of environmental gradients on body shape of *M. s. splendida* within the Daintree rainforest, we adapted the RDA approach used for the GEAs (described above) to implement phenotype-environment analyses (PEAs). We used the same set of environmental explanatory variables (above), this time testing body shapes (PCs of individual Procrustes distances determined as significant by Broken-Stick method) as response variables. We again controlled for putatively neutral genetic structure (allelic covariance Ω; pairwise *F*_ST_; waterway distance), plus the additional covariable of body size (log centroid size). Inputs for the body shape response variable and size covariable were created in R, using functions developed by Claude (2008) (full details in S1j).

### Genotype-phenotype-environment analysis

If environmental selection for a particular phenotype has promoted evolutionary adaption, then the relevant phenotypic divergence should be accompanied by a genotypic response. We therefore tested whether any of the putative adaptive (environmentally associated) genetic variation could be attributed to environmentally associated morphological variation throughout the study region. This could indicate both a heritable component to the associated body shape traits (as opposed to the alternative hypothesis of phenotypic plasticity), as well as provide further support for their adaptive advantages. In R, we ran a global RDA using the four significant PCs of individual Procrustes distances as explanatory variables, and 864 putative adaptive alleles (identified in the genotype-environment RDA controlling for Ω) as the multivariate response. The analysis was then repeated as a partial RDA using individual body size (log centroid) as a covariable (details S1k), the results of which isolated only the genotype-phenotype interactions best explained by environmental selection.

### Functional annotation

From the *M. duboulayi* reference genome (Beheregaray et al. unpublished data; Brauer et al. 2023), we extracted 300 bp flanking sequences on either side of the same 864 candidate SNPs. These were aligned to the UniProtKB/Swiss-Prot database using BLASTx (BLAST + 2.12.0; Camacho et al. 2009). Matches with e-value < = 1e-3 were annotated with gene ontology (GO) terms and associated functional descriptions.

## Results

### Genome-wide SNP data, diversity, and population structure

**Fig. 3:**
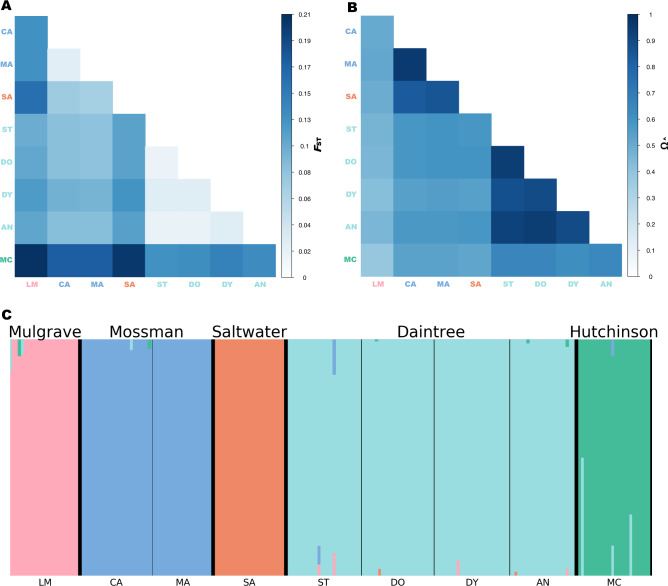
Genomic differentiation and population structure of the eastern rainbowfish *Melanotaenia splendida splendida*. **A** Heatmap of pairwise F_ST_ based on 14,478 putatively neutral SNPs; **B** Correlation map for BAYPASS core model scaled covariance matrix Ω based on allele frequencies of the full dataset of 14,540 SNPs; and **C** Cluster plot based on FASTSTRUCTURE analysis of 14,478 putatively neutral SNPs, where colours represent inferred ancestral populations of individuals based on an optimal *K* of five. Large type refers to drainage systems, which are separated by thicker black lines. Small type refers to sampling localities, separated by thinner black lines. Locality abbreviations follow Table 1.

Sequencing produced ~550 million ddRAD reads for 242 *M. s. splendida* individuals (including replicates). After variant filtering and removal of lower-quality samples, we retained 14,540 putatively unlinked SNPs (Table S2a), of which 14,478 could be considered neutral for the purposes of population genomic analyses (Fig. S2a). The final dataset comprised 210 high-quality individuals across nine sampling sites. Neutral genomic diversity (Table 1) was moderately high for most sites, with expected heterozygosity (*H*_E_) ranging from 0.278 to 0.321 (mean = 0.293), and proportion of polymorphic loci (*PP*) ranging from 0.252 to 0.391 (mean = 0.329). Population subdivision accounted for a substantial proportion of the neutral variation, with global *F*_ST_ = 0.165, and *F*_IT_ = 0.205. None of the site-specific *F*_IS_ values (Table 1) were significant. Pairwise *F*_ST_ comparisons (Fig. 3a; Table S2b) indicated relatively little differentiation between localities within the same drainage (0.017–0.029; mean = 0.024) compared with localities in different drainages (0.071–0.208; mean = 0.120), consistent with a segregating effect of drainage boundaries. Similarly, greater correlations in allelic covariance (Fig. 3b) were observed among, rather than within drainages. Both pairwise and site-specific *F*_ST_ values indicated that the most neutrally divergent sampling localities were the northernmost McClean Creek (Hutchinson Drainage), followed by the more centrally located Saltwater Creek (Saltwater Creek Drainage). In addition to being the smallest drainage systems sampled, both are located along the coastal boundary of the species distribution (Fig. 1).

**Table 1:** Genetic diversity measures and *F*-statistics for the eastern rainbowfish *Melanotaenia splendida splendida* at nine rainforest localities, based on 14,478 putatively neutral loci.

Location	Site Code	Drainage system	*n*	*H*_E_	*H*_O_	*PP*	*F*I_S_	*F*_ST_
Little Mulgrave Creek	LM	Mulgrave	23	0.283	0.271	0.323	0.018	0.204
Cassowary Creek	CA	Mossman	23	0.297	0.295	0.314	−0.011	0.177
Marrs Creek	MA	Mossman	20	0.307	0.293	0.305	0.019	0.178
Saltwater Creek	SA	Saltwater Creek	24	0.321	0.307	0.264	0.019	0.261
Stewart Creek	ST	Daintree	25	0.278	0.259	0.391	0.031	0.065
Douglas Creek	DO	Daintree	24	0.289	0.272	0.376	0.038	0.060
Doyle Creek	DY	Daintree	24	0.294	0.280	0.358	0.030	0.095
Forest Creek	AN	Daintree	22	0.289	0.268	0.377	0.054	0.059
McClean Creek	MC	Hutchinson	25	0.279	0.271	0.252	0.009	0.382

*n* sample size for final DNA dataset, *H*_E_ expected heterozygosity, *H*_O_ observed heterozygosity, *PP* proportion of polymorphic loci, *F*_IS_ site-specific inbreeding coefficient, *F*_ST_ site-specific *F*_ST_.

Low differentiation within drainages and high differentiation among drainages was also reflected by clustering analyses. Both FASTSTRUCTURE (Fig. 3c) and DAPC (Fig. S2c) grouped individuals by their drainage system of origin, resulting in an optimal *K* of five for both analyses. Pairs of drainages in relatively close geographic proximity (i.e., Daintree and Hutchinson; Saltwater and Mossman) grouped more closely in the DAPC, indicating similarities in genetic variation which may result from a more recent shared history. The neighbour-joining tree (Fig. S2d), representing putative individual-level evolutionary relationships, presented each drainage system as reciprocally monophyletic and supported a hierarchical pattern of spatial connectivity. Contemporary gene flow among drainages was very low, with inferred migration rates (m) ranging from 0.0033–0.0224 between the inferred populations, versus 0.9521–0.9865 within the populations (Fig. S2e, Table S2e).

### Genotype-environment associations

**Fig. 4:**
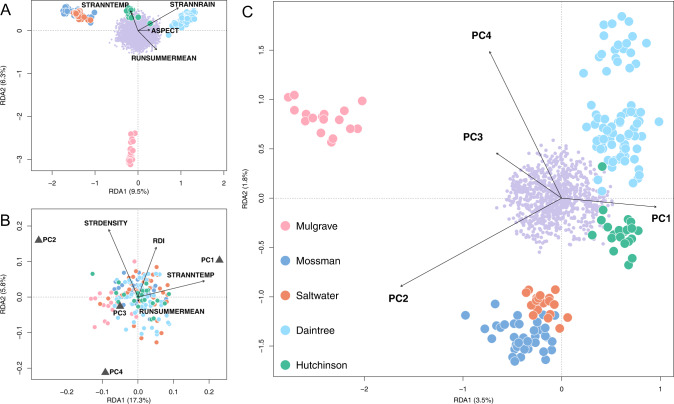
Ordination plots summarising the first two axes of partial redundancy analyses (pRDAs) for *Melanotaenia splendida splendida* individuals sampled across nine rainforest sampling localities in the Wet Tropics of Queensland. **A** Genomic variation (based on 14,540 SNPs) explained by five associated environmental variables, after partialing out the locality-specific effect of Ω (allelic covariance). Environmental predictors accounted for 16.6% of total variation (*p* = <0.001). **B** Body shape variation (based on 18 morphometric landmarks) explained by four associated environmental variables, after partialing out the locality-specific effect of Ω (allelic covariance) and the individual effect of body size (log centroid). Environmental predictors accounted for 14% of total variation (*p* = <0.001). **C** Genomic variation (based on 864 putative climate-adaptive alleles) explained by four associated principal components of body shape, after partialing out the individual effect of body size (log centroid). Body shape accounted for 6.5% of climate-associated genetic variation (*p* = <0.001). For all plots, large points represent individual-level responses, and are coloured by drainage system of origin. Small purple points represent SNP-level responses. Vectors represent the magnitude and direction of relationships with explanatory variables.

Without considering neutral influences, global redundancy analyses (RDAs) found six environmental variables associated with 23% of the observed genetic variation among individuals (*p* = <0.001; Fig. S2f). After controlling for locality-specific neutral variation, GEAs remained highly significant (*p* = <0.001). Controlling for scaled allelic covariance Ω (Figs. 4a; S2e), associations with five environmental variables accounted for 16.6% of total SNP variation, from which 864 loci were identified as candidates for environmental selection (*p* ≤ 0.0027; Fig. S2g). The environmental explanatory variables STRANNRAIN and STRANNTEMP were the most influential in the model. When controlling for the alternative neutral covariable of pairwise *F*_ST_ (Fig. S2e, h), associations with six environmental variables accounted for 12.1% of total SNP variation, with STRANNRAIN and STRANNTEMP likewise emerging as the most influential. When controlling for the neutral proxy of waterway distance (Fig. S2e, i), associations with six environmental variables accounted for 15.7% of SNP variation, in this instance with STRANNTEMP and ASPECT as the most influential. However, STRANNRAIN and STRANNTEMP were once again the most important in the BAYPASS GEA approach (auxiliary covariate model; Fig. S2j), which identified a more conservative 176 loci as candidates. Of these, 88 were uniquely associated with STRANNRAIN, 56 with STRANNTEMP, 12 with ASPECT, ten with RDI, nine with STRDESITY, and one with RUNSUMMERMEAN. Twenty percent of these candidates (36 loci) were shared with the pRDA approach.

### Morphological variation among localities and environmental gradients

Across the sampled range of rainforest *M. s. splendida*, four PCs of body shape (Figs. 2b, S2k) were identified as significant by Broken-Stick modelling. Major shape changes along these axes included differences in body depth (PCs 1 and 4), dorsal and ventral curvature (PCs 2 and 3), fin length and position (PCs 3 and 4), and upturn of head and mouth (PCs 2 and 3). Despite some overlap of individual variation among localities, CVAs revealed significant differences (*p* < 0.05) in Procrustes distances among most sampling sites, and among all drainages/populations (Fig. S2l, Table S2l). Interestingly, the sites for which shape difference could not be significantly distinguished (Forest Creek, Daintree drainage; and McClean Creek, Hutchinson drainage) were not within the same drainage system (or neutrally inferred population grouping) but were the closest sites in geographical proximity. The most shape-divergent localities were Little Mulgrave (Mulgrave drainage) and Doyle Creek (Daintree drainage).

Global RDAs found that ~24% of body shape variation (based on four significant shape PCs) was associated with environment (*p* = <0.001; Fig. S2m). After controlling for possible allometric (log centroid size) and neutral genetic (locality-specific allelic covariance Ω) influences using pRDA, 14% of body shape variation remained significantly associated with four environmental variables, with STRANNTEMP and STRDENSITY the most influential (*p* = <0.001; Figs. 4b; S2e). The body shape components most strongly associated with environment were PC2, relating to dorsal flattening, ventral curvature, and upturn of head; and PC4, relating to width and position of first and second dorsal fins and anal fin, body depth, and length of caudal peduncle (see Fig. 2 for graphical representation). pRDAs controlling for alternative covariables of pairwise *F*_ST_ and waterway distance (each together with body size), respectively found 11.9% and 11.6% of shape variation associated with environment (Fig. S2e, n, o).

### Associations among genotype, phenotype, and environment

The GxPxE analysis using global RDA revealed a significant association (*p* = <0.001) between putatively adaptive genetic variation and morphology, to which 6.8% of divergence in those loci could be attributed. After controlling for possible allometric effects (centroid size) using pRDA, this figure was only slightly reduced to 6.5% (Fig. 4c). The PCs of body shape that had the strongest influence on the model were PC2, followed by PC4. Based on these associations, we identified 61 candidate loci for climate-adaptive morphological variation with *p* = <0.0455 (Fig. S2p). In other words, these loci are predicted to confer a heritable selective advantage under localized environmental conditions based on their association with body shape.

### BLAST annotation results

Of 864 candidate adaptive loci, 128 were successfully aligned to protein sequences in the UniProtKB/Swiss-Prot database and annotated with GO terms (Table S2q). Five of these were also identified as candidates by the GxPxE analysis: cdh2, farsa, Fbxl3, MON1B, SPTAN1.

## Discussion

As some of the most diverse, iconic, and potentially vulnerable ecosystems in the world, tropical rainforests remain remarkably understudied. Their complex and often inaccessible nature has created ongoing challenges to identifying the processes which drive and maintain biodiversity. Here, we contribute insight into these processes on an intraspecies level by addressing influences on genetic and morphological variation across the rainforest range of an Australian tropical fish (*Melanotaenia splendida splendida*). A clear association was found between both genetic and morphological variation and the drainage divisions of this highly structured catchment system, indicating an important role of gene flow limitations on population divergence. Despite this, a larger component of divergence was better explained by local environmental conditions, and especially by variables relating to hydroclimate. This pattern was particularly pronounced for the morphological component of diversity, providing further evidence for its functional relevance. Meanwhile, GxPxE associations identified highly significant relationships between major components of body shape divergence and ecologically associated genetic variants. Based on these consistencies, we propose that local evolutionary adaptation is a favourable contributor to the high phenotypic diversity renowned of *M. s. splendida* (Pusey et al. 2004). We also infer that hydroclimatic adaptation has been a central mechanism for local divergence in this species, posing future challenges under rapid climatic change.

### Environmental selection as a driver of rainforest freshwater diversity

Although there has been substantial historical emphasis on vicariant drivers of tropical rainforest diversity, an increasing number of genomic studies have revealed a dominant influence of contemporary environment (Ntie et al. 2017; Termignoni‐García et al. 2017; Zhen et al. 2017; Lam et al. 2018; Jaffé et al. 2019; Miller et al. 2020; Morgan et al. 2020). Most of these works have focussed on terrestrial species, finding strong associations with either temperature or precipitation. In the Wet Tropics of Queensland, hydroclimatic variations dependent on latitude, elevation, terrain, and human impacts (Metcalfe and Ford 2009; Terrain NRM 2016) mean that hydroclimatic selection could be expected to contribute to geographic patterns of diversity in freshwaters. Consistent with this hypothesis, we found strong evidence for environmental influences on both genetic and body shape divergence of *M. s. splendida*, even after accounting for approximations of neutral demographic structure. Highly significant genotype-environment associations (GEAs) were supported by both RDA and BAYPASS analytical approaches. Depending on the covariables included, partial RDAs attributed ~12–17% of allelic variation to associations with key environmental variables, in contrast to the ~10–15% of variation which could be equally well or better explained by neutral conditional variables. Although it is difficult to draw direct comparisons, such strong GEAs support, and even exceed, those previously described for related temperate and subtropical Australian rainbowfishes (*M. fluviatilis*, Brauer et al. 2018; *M. duboulayi*, Smith et al. 2020).

Large associations with environment were also found between body shape and environment in phenotype-environment associations (PEAs), with greater overlap among sites indicating that morphology may be more conserved than genotype. Environment accounted for ~7–14% of body shape variation in partial RDAs after accounting for conditional variables of neutral genetic structure and centroid size. These conditional variables accounted for a much larger 44–50% of shape variation, but intriguingly, most of this related to a large effect of size rather than of neutral genetic structure, which could only explain ~4% of shape variation alone. In contrast to the relatively large contribution of neutral structure in the GEAs, this pattern was surprising, yet plausible, under the premise of greater functional constraints on morphology than on genome-wide variation. While many genomic changes may have little functional relevance (e.g., synonymous substitutions, pseudogenes, noncoding sequences), it has been suggested that the effects of random drift on phenotypes, and particularly on morphology, are less likely to be truly neutral (Ho et al. 2017; Zhang 2018; but see Wideman et al. 2019). That is, if a physiological trait is subject to strong selection (directional or otherwise), it is unlikely to conform to neutral patterns unless genetic drift is also extremely strong (McKay et al. 2001; Clegg et al. 2002). Considering that body shape variation in teleosts has well-established roles related to swimming biomechanics, sensory ability, sexual behaviour, and various life history traits (Hanson and Cooke 2009; Langerhans and Reznick 2010; Killen et al. 2016), it is congruous that only a small proportion of variation would be explained by demography.

Few studies in the tropics have so far attempted to link signals of local genetic adaptation with patterns of phenotypic divergence. However, notable overlaps in genetic and morphological associations with environment have been detected by Morgan et al. (2020) for the rodent *Praomys misonnei* in relation to precipitation and vegetation structure, and by Miller et al. (2020) for the frog *Phrynobatrachus auritus* in relation to seasonality of precipitation. Here, we found a strong association between 6.5% of environmentally associated genetic loci and body shape PCs. While the relationship between these variables remains putative, a plausible explanation is that genes linked to the 61 implicated loci are contributing to body shape differences among sampled sites. Although only five of these loci were annotated to protein sequences, two have known phenotypic relevance in zebrafish (*Danio rerio*), including cdh2 (Cadherin-2) and SPTAN1 (Spectrin alpha chain, non-erythrocytic 1). Cdh2 is involved in early development (morphogenesis; Tay et al. 2010), while SPTAN1 is expressed in the fin, eye, cranial ganglion, and post-vent region (Farnsworth et al. 2021), with knockout affecting motor nerve formation (Voas et al. 2007; Susuki et al. 2011). These findings could suggest a heritable component of phenotypic diversity in *M. s. splendida*, congruent with previous evidence for the heritability of rainbowfishes’ hydrodynamic morphology (*M. eachamensis*; McGuigan et al. 2003) and transgenerational heritability of transcriptional plasticity (*M. duboulayi*, McCairns et al. 2016). In the former example, similar phenotypic differences linked to hydrology were maintained by offspring produced in a common garden environment, providing evidence for evolved functional differences. The association of these signals thus adds an additional layer of support for the influence of local environment on evolutionary trajectories in the Wet Tropics.

In considering which environmental variables may have been the most influential in shaping diversity, repeated associations with thermal and hydrological variables indicated a strong role for hydroclimate. Average annual rainfall and average annual temperature were the strongest environmental predictors of genotype regardless of the GEA software, statistical approach, or neutral covariable used. The PEAs also emphasised the role of hydroclimate, with average annual temperature and stream density explaining the greatest shape variation. As with the GEAs, average annual rainfall was strongly associated with body shape in global RDA modelling. However, its covariation with body size meant effects could not be reliably separated from the alternative hypothesis of allometric shape change. Regardless, both GEA and PEA results accord with globally applicable expectations for climate as a driver of functional diversity (Hawkins et al. 2003; Siepielski et al. 2017), and emerging evidence for its importance in terrestrial tropical adaptation. As is common with environmental association, it is difficult to determine whether tested environmental variables are directly affecting studied organisms’ physiologies, or are interacting via affiliated but unmeasured environmental factors (Rellstab et al. 2015). However, even indirect associations with abiotic variables can reflect connected effects at other ecological levels (Blois et al. 2013). Thermal and hydrological associations in this freshwater context support the evolutionary relevance of climatic variance to wet tropical diversity, a key finding in light of the ‘ecology vs isolation’ debate.

### Putative trait adaptation to local environment

Body shape may be one of the best indicators of a fish’s inhabited niche (Gatz Jr 1979; Wainwright 1996; Shuai et al. 2018), and shape changes with important associations in this system match several well-described physiological adaptations in other teleosts, including rainbowfishes (McGuigan et al. 2003; McGuigan et al. 2005; Smith et al. 2020). Here, shape PCs 4 and 2 had the strongest relationship with environmentally associated alleles, making them among the most likely to have a heritable adaptive relevance. Interestingly, PC4 was mostly characterised by a change in fin positions, with some striking similarities to those described by McGuigan et al. (2003) and McGuigan et al. (2005) for congeneric *M. duboulayi* and *M. eachamensis*. These studies found that across lineages, streamflow conditions were consistently associated with insertion points of the first dorsal and pelvic fins, as well as the width of the second dorsal fin base. Here, changes on PC4 similarly included insertion of the first dorsal fin and width of the second dorsal fin base, and the associated precipitation and stream density variables can be related directly to stream flow (Carlston 1963).

Shape change on PC2 was not only relevant in the GxPxE analyses but was also the most important shape variable directly associated with environment (PEAs). Positive values coincided with a more upturned head, smaller eye, reduced dorsal hump, and distended pelvic region. Much of this divergence appeared latitudinally, with upturned shape extremes more common in the higher rainfall northerly catchments of Hutchinson, Daintree, and Saltwater. In a variety of teleost species, an upturned head and flattened dorsal region have been associated with a tendency for surface dwelling and feeding (Wootton 2012), surface breathing in oxygen-deficient waters (Lewis Jr 1970; Kramer and McClure 1982), and predation intensity (Langerhans et al. 2004; Eklöv and Svanbäck 2006). While an arching body shape has also been associated with rigor mortis in fishes (Hooker et al. 2016), the immediate imaging of individuals at the time of death, consistent among sampling sites, is likely to have prevented locality-specific differences. *M. s. splendida* is known for an omnivorous feeding strategy, including floating material such as invertebrates (Pusey et al. 2004). Notably, the surface-feeding tendency of the related *M. duboulayi* has been associated with differences in vegetative cover, possibly due to thermoregulatory influences or predator density (Hattori and Warburton 2003). Therefore, while this component of shape variation could be explained by a variety of factors, promising hypotheses include local selective differences due to relative abundance of food sources, predator presence, or vegetation structure.

In addition to the described adaptive signals occurring throughout the region, our results suggested an important effect of drainage structure in demographic divergence. Both demographic and environmental association analyses indicated gene flow barriers across contemporary drainage boundaries, delineating populations and affecting broader patterns of diversity. This was reflected by low inter-drainage migration rates and genetic clustering within drainages. Some additional, shallow substructure was detected among sampling sites within drainages, possibly resulting from isolation by distance or other resistance within the stream network. Similar hierarchical configurations have been previously described for subtropical and temperate rainbowfishes (*M. fluviatilis*, Brauer et al. 2018; *M. duboulayi*, Smith et al. 2020), reflecting a recognised pattern of connectivity in lotic environments (Grummer et al. 2019). We therefore propose that, in addition to hydroclimatic factors, the geographic arrangement and relative size of individual watersheds has promoted evolutionary divergence.

### Considerations for the ongoing maintenance of adaptive diversity in tropical rainforests

Both the strong effects of hydroclimate on intraspecies diversity, and the geographical confinement created by catchment structure, indicate that climate warming could place strong selective pressure on rainforest populations of *M. s. splendida*. Climate projections for the Wet Tropics cluster region indicate increases in average temperature, as well as more extreme weather events including rainfall intensity (Hilbert et al. 2014). If a large component of local diversity has developed in either a direct or indirect response to climate, we can expect that alteration of current environmental conditions will necessitate an adaptive response (Fitzpatrick and Keller 2015; Bay et al. 2017). It is notable that signals of adaptive divergence were directionally similar for genotype and morphology, and significant overlaps were revealed by GxPxE results. But as previously discussed, there were also some differences among associated environmental variables, their respective contributions, and the relative influences of neutral processes. These factors suggest similar but non-identical ecological dynamics are contributing to genetic and morphological diversity across the studied riverscapes. It therefore seems likely that while management strategies informed by either component of diversity should produce common benefits, a knowledge of both components would benefit more comprehensive management.

*Melanotaenia splendida splendida* is one of the most abundant fishes in the Queensland Wet Tropics (Pusey et al. 2004), and our results indicated relatively high genetic variation in most populations. Moreover, the total species range extends beyond rainforest limits (ALA 2020). Hydroclimate-associated variation in this species may highlight the need for further adaptation in response to climate change; but, perhaps more concerning are the implications for already vulnerable tropical freshwater species. Species with small effective population sizes and low genetic diversity are likely to have less standing variation available for selection (Frankham 2015; Ralls et al. 2018), and opportunities for future adaptation have a greater chance of being outweighed by random genetic drift (Perrier et al. 2017). While not all tropical rainforests exhibit as structured terrain as the Queensland Wet Tropics, mountainous features are common to most continental tropics. Moreover, rainforests are becoming globally affected by less predictable flow dynamics (Jiménez-Cisneros et al. 2014) and accumulating human modifications (Davis et al. 2018). In the context of dendritic systems, even relatively small structural changes can divide the habitat area over which gene flow can occur (Davis et al. 2018; Blanchet et al. 2020). We therefore suggest that the maintenance of existing connectivity should be prioritised in tropical rainforest river networks, and support a proactive strategy of evolutionary rescue for particularly vulnerable taxa (*sensu* Ralls et al. 2018).

## Conclusion

Our work indicates that the interplay between contemporary hydroclimatic variation and drainage connectivity has helped shape regional diversity in the tropical rainforest fish *M. s. splendida*. Consistent with a growing body of work in Australian rainbowfishes, both genomic and morphological divergence appeared relevant to local adaptation, including heritable and hydrologically associated traits identified in related species. Moreover, three-way associations detected among genotype, phenotype, and environment supported the possibility of heritable, climate-adaptive shape variation in *M. s. splendida*. Empirical evidence for the role of temperature and precipitation driving phenotypic divergence has been mounting in tropical rainforest research, however this is likely the first freshwater example to benefit from a high-resolution genomic dataset. Where possible, we suggest that future work should continue to integrate environmental, genomic, and phenotypic datasets to disentangle evolutionary processes applicable to both conservation and theoretical development.

### Data archiving

The relevant data have been appropriately archived and are available at figshare: https://figshare.com/s/c4b75d40fea91ab38beb. Data will be made publicly available upon acceptance.

## Supplementary information

Supplemental Material
