# Supplementary material for: Environmental selection, rather than neutral processes, best explain regional patterns of diversity in a tropical rainforest fish
Source: Heredity (Edinb). 2023 Mar 30;130(6):368–80. doi: 10.1038/s41437-023-00612-x (PMC10238429; doi:10.1038/s41437-023-00612-x)
Supplement: Supplementary file 1 — Supplemental Material [file 41437_2023_612_MOESM1_ESM.docx]

Supplementary information for ‘Environmental selection, rather than neutral processes, best explain regional patterns of diversity in a tropical rainforest fish’

Part 1: Supplementary Methods; Part 2: Supplementary Results

S1. Supplementary Methods

*S1a. Sample collection*

*Melanotaenia splendida splendida* (eastern rainbowfish) were sampled from nine rainforest creek sites across five drainages in the Wet Tropics of Queensland, north-eastern Australia. To photograph individuals for morphometric data collection, each was positioned on a polystyrene tray immediately after death, submerged in a shallow layer of water to prevent distortion of shape by bending. Dissection pins were used to display the fish in a standard orientation (right-side-down) and to fix fins into their expanded state. Specimens were photographed using a Canon EOS 6D DSLR (EF-S 35mm f2/2.8 macro lens) attached to a horizontal mount positioned 45 cm directly above the specimens, and a ruler was included in each photograph for scaling.

Table S1a. Localities and sample sizes (n) of *Melanotaenia splendida splendida* collected from the Wet Tropics of Queensland for genomic DNA and morphometric data.

| Location | Catchment | Latitude | Longitude | Collection date | Collected *n* | Final n (DNA) | Final n (Morpho) | Final n (GxPxE) |
| --- | --- | --- | --- | --- | --- | --- | --- | --- |
| Little Mulgrave Creek | Mulgrave | -17.13 | 145.7 | 31/03/2017 | 30 | 23 | 20 | 17 |
| Cassowary Creek | Mossman | -16.51 | 145.41 | 23/03/2017 | 30 | 23 | 30 | 23 |
| Marrs Creek | Mossman | -16.47 | 145.36 | 23/03/2017 | 24 | 20 | 19 | 15 |
| Saltwater Creek | Saltwater Creek | -16.42 | 145.36 | 23/03/2017 | 30 | 24 | 21 | 19 |
| Stewart Creek | Daintree | -16.32 | 145.32 | 23/03/2017 | 30 | 25 | 22 | 20 |
| Douglas Creek | Daintree | -16.28 | 145.3 | 23/03/2017 | 30 | 24 | 29 | 21 |
| Doyle Creek | Daintree | -16.26 | 145.45 | 24/03/2017 | 30 | 24 | 23 | 22 |
| Forest Creek | Daintree | -16.25 | 145.39 | 24/03/2017 | 31 | 22 | 21 | 18 |
| McClean Creek | Hutchinson | -16.23 | 145.42 | 24/03/2017 | 32 | 25 | 22 | 22 |

*S1b. DNA extraction*

For DNA extractions by salting-out (modified from Sunnucks and Hales (1996), we placed approximately 5 mm^2^ of each fin sample (crushed) in individual 1.5 mL microfuge tubes with 600 μL extraction buffer TNES, 20 μL proteinase K (10 μg/μL) and 10 μL RNase (10 μg/μL). Tubes were incubated at 37°C for three hours before adding 70 μL ammonium acetate, shaking for 15 seconds, chilling at -80°C for 5 minutes and centrifuging at 14,000 rpm for 5 minutes to precipitate proteins. Supernatant was decanted into a new 1.5 mL tube with 1 mL 99% ethanol, chilled at -80°C for 5 minutes, and centrifuged at 14,000 rpm for 5 minutes to precipitate DNA. Ethanol was removed and the DNA pellet was washed twice with 70% ethanol solution. The pellet was air-dried and resuspended in 17 μL of TE buffer. High-quality samples were diluted to 20 ng/μL and stored at -20°C.

*S1c. Library preparation*

For each sample, 300 ng of genomic DNA was digested with SbfI-HF and MseI restriction enzymes (New England Biolabs). The cleaved fragments were ligated to adapter sequences and one of 96 unique 6-bp barcodes designed in-house. Groups of 12 individual samples were then pooled to create 8 libraries per lane and purified using AMPure XP beads (Agencourt) to remove small DNA fragments and other contaminants. Then, DNA size-selection was performed using automated gel electrophoresis (agarose, 1.5%) via Pippin Prep (Sage Science) to select fragments within a 250 – 800 bp range. A Qubit fluorometer (Life Technologies) was used to quantify library concentrations. Finally, libraries were amplified by polymerase chain reaction (PCR), using two 25 μL reactions per pool to minimise PCR clonal artefacts associated with larger volumes. Reactions were recombined, and a 2100 Bioanalyzer (Agilent Technologies) was used to verify that fragment size distribution was within the target range. Both the Qubit fluorometer (Life Technologies) and Real Time PCR were used to reconfirm quantity of DNA, and each of the 8 libraries were pooled in equimolar concentrations to form five lanes of 96 uniquely barcoded samples.

*S1d. Bioinformatics: read trimming, alignment to genome, variant calling and filtering*

Using _VCFTOOLS_ 0.1.15 (Danecek et al. 2011), we removed loci with >20% missing data and minor allele frequency <3%, with the latter being biologically feasible but commonly related to calling errors. We also removed loci within indels, which can arise by different mechanisms and produce different functional effects than SNPs. We checked frequency of missing data per individual, and from the original unfiltered dataset, removed individuals with >30% missing data. The above filtering steps were then repeated for the unfiltered dataset with low coverage individuals removed to produce a filtration unbiased by low quality samples.

Also using _VCFTOOLS_, complex genotypes (e.g., multi-nucleotide polymorphisms) were decomposed and removed. We filtered by quality, compensating by coverage (QUAL / DP > 0.20) to prevent unrealistic inflation of locus quality scores (Li 2014). We removed loci with mapping quality >30, then calculated the mean depth of coverage and filtered by the mean +2SD to remove potentially merged paralogous sites. We also filtered for Hardy Weinberg Equilibrium (HWE) by sampling location, removing SNPs < *p* = 0.05 in 25% or more populations. Although large deviations from HWE are expected among populations due to non-random mating, these deviations can indicate erroneous variant calls when occurring within sampling sites.

Finally, we implemented a filter for linkage disequilibrium (LD) to reduce the likelihood of non-random associations among loci due to proximity in the genome. We first used _VCFTOOLS_ to calculate the correlation coefficient between each pair of loci. In _R_ (RC Team 2019), we fitted a spline to calculate the exponential decay of LD by physical distance (bp) and used a Tukey anomaly criteria (95% probability distribution; ) to select a cut-off (189 bp) where the rate of linkage decay was no longer significant. Given that R^2^ values (and therefore LD) do not statistically decrease beyond this distance, most SNPs are expected to be unlinked. Where more than one of the identified SNPs occurred within the cut-off distance, all but one were excluded from the dataset. This left a total of 14,540 high quality SNPs for further analysis.

*S1e. Differentiating putatively neutral versus outlier loci*

Prior to assessing conformity of loci to neutral expectations we ran a preliminary structure analysis using _FASTSTRUCTURE_ 1.0 (Raj et al. 2014) for the full filtered dataset of 14,540 SNPs. We first converted the VCF file to _FASTSTRUCTURE_ format using _PGDSPIDER_ 2.0 (Lischer and Excoffier 2012), then ran the model with the default convergence criterion of 10^−6^, a simple prior, and ten replicate runs per a maximum of 10 *K*. The number of model components best able to explain structure in the data was determined using the function “chooseK.py”.

*S1f. Genetic diversity and inference of population structure*

To prepare input files for population genetic analyses, we converted the full SNP dataset and putatively neutral dataset from VCF to STRUCTURE (.str) format using _PGDSPIDER_ . The same program was used to subsequently convert STRUCTURE files to FASTSTRUCTURE (.str), ARLEQUIN (.arl) and PAUP* (concatenated SNPs; phylip format) formats. For _BAYPASS_ 2.2 (Gautier 2015), _PGDSPIDER_ was first used to convert .str files to GESTE format, before using the script *geste2baypass* (Pina-Martins 2016) to create a _BAYPASS_ (.txt) file with allele counts based on sampling locality. For packages implemented in _R_ (e.g. _ADEGENET_, _HIERFSTAT_, _VEGAN_, and others), .str files were imported as GENIND objects using _ADEGENET_ 2.0.0 (Jombart 2008).

To produce an unrooted Neighbour Joining Tree, we imported the neutral SNP dataset in concatenated (phylip) format to _PAUP*_ 4.0 (Swofford and Sullivan 2003). We ran the Neighbour Joining Tree analysis using pairwise TN93 distances (Tamura and Nei 1993), with other settings as default. *N.B.* where one individual was identified as an extreme outlier, photographic documentation was re-examined to confirm species identification error. The misidentified individual, confirmed as a co-distributed but non-hybridising *Melanotaenia maccullochi*, was removed from subsequent analyses, and prior population genetic analyses were repeated.

To produce a scaled covariance matrix of population allele frequencies (Ω), we used _BAYPASS_ 2.2 (Gautier 2015) core model, based on the full SNP dataset. This hierarchical Bayesian model explicitly incorporates neutral correlation structure, providing an informative basis for demographic inference by accounting for structure resulting from shared history. The method follows from the BayEnv model proposed by (Coop et al. 2010, Günther and Coop 2013), but with several extensions to improve accuracy by estimation of prior distributions. The core model was executed using the command line, with default settings. From here, the resulting scaled covariance matrix (Ω) was visualised in R, using the *cov2cor* R function to produce a correlation matrix ∑, which was plotted as a correlation heatmap.

Using the neutral dataset, we re-examined population structure using _FASTSTRUCTURE_ 1.0 (Raj et al. 2014), an algorithm for variational Bayesian inference of global ancestry. This method assesses allele frequency variations to find the number of clusters best approximating the log-marginal likelihood of parametric posterior distributions over hidden variables. We ran the model with the default convergence criterion of 10^−6^, a simple prior, and ten replicate runs per a maximum of 10 *K*. The most likely number of clusters was selected using the function *chooseK*, and visualised using _DISTRUCT_ 1.1 (Rosenberg 2004). We then used a Discriminant Analysis of Principal Components (DAPC) in _R_ package _ADEGENET_ to independently identify and describe the optimal number of genetic clusters present. DAPC considers both between- and within-group variance to best describe differences between groups, while minimising variation within. The function *find.clusters* was first used to transform the data using PCA, and then to run a *k-*means algorithm with increasing values of *k* (up to a possible 9 *k*, the number of rainforest sampling sites) using all PCs.

To estimate migration rates using BA3-SNPS 1.1 (Wilson and Rannala 2003, Mussmann et al. 2019), we used the putatively neutral unlinked SNP dataset, which was divided randomly among four separate runs to reduce the total run time. Each run was performed with 10 million iterations plus one million iterations as burn-in. Mixing parameters (allele frequencies, inbreeding coefficients, and migration rates) were adjusted to achieve the acceptance rates recommended by Wilson and Rannala (2003). Average m values from the four runs were plotted using Circlize 0.4.15 (Gu et al. 2014).

*S1g. Characterising environmental variation*

National Environmental Stream Attributes v1.1.3 were obtained Geoscience Australia (Stein 2011), a custodian for national surface hydrology data. The National Environmental Stream Attributes describe both natural and anthropogenic characteristics of the stream and catchment environment supplied by state and national jurisdictions to form a comprehensive national dataset. We initially downloaded lookup tables for all available attributes (>400) and, using _ARCMAP_ 10.3 (ESRI 2011), connected the relevant attributes for each sampling site using raster files from the associated 9 Second DEM Derived Stream Network. Of the available variables, we pruned those for which there was no variation between sampling sites, were provided at a scale larger than the distance between most sampling sites (i.e. catchment level as opposed to stream level), or had missing data for any of the sampling sites. After this, ~83 variables remained. A Pearson correlation was performed in _R_, and if two attributes were highly correlated (|r| ≥ 0.7), one was removed from the dataset. While we recognise that there is not a perfect way of selecting which variables to keep, particularly where variables interact with each other, we prioritised retention of variables considered less likely to be derived in the system, and most likely to be important for the biology of the species, as indicated in previous studies of Australian freshwater fishes (e.g. Attard et al. (2018), Brauer et al. (2018)).

Table S1g. Details of National Environmental Stream Attributes v1.1.3 (Stein 2011), considered for environmental association analyses of rainforest populations of *Melanotaenia splendida splendida.*

| Variable | Attribute | Units | Description |
| --- | --- | --- | --- |
| ASPECT | stream segment aspect | ° | Mean aspect of the stream segment grid cells (computed by taking the mean of the northerly and easterly components of the direction of flow separately) |
| RDI | river disturbance index | Index 0-1 | River Disturbance Indices and Factors derived using the method of Stein, J. L., Stein, J. A. and Nix, H. A. (2002) Spatial analysis of anthropogenic river disturbance at regional and continental scales: identifying the wild rivers of Australia. Landscape and Urban Planning, 60, 1-25 |
| RUNSUMMERMEAN | average summer mean runoff | ML | Mean of the totals for summer for the years 1971-2000 for the monthly accumulated soil water surplus values at the stream segment pour-point |
| STRANNRAIN | average annual mean rainfall | mm | Average value of BIOCLIM parameter "Annual mean rainfall" of all grid cells comprising the stream segment and associated valley bottoms |
| STRANNTEMP | average annual mean temperature | °C | Average value of BIOCLIM parameter "Annual mean Temperature" of all grid cells comprising the stream segment and associated valley bottoms |
| STRDENSITY | total length of upstream segments calculated for the segment pour-point | km/km^2 | Total segment length (RCHLEN) of all upstream segments in the DEM derived stream network / contributing area calculated for the segment pour-point |


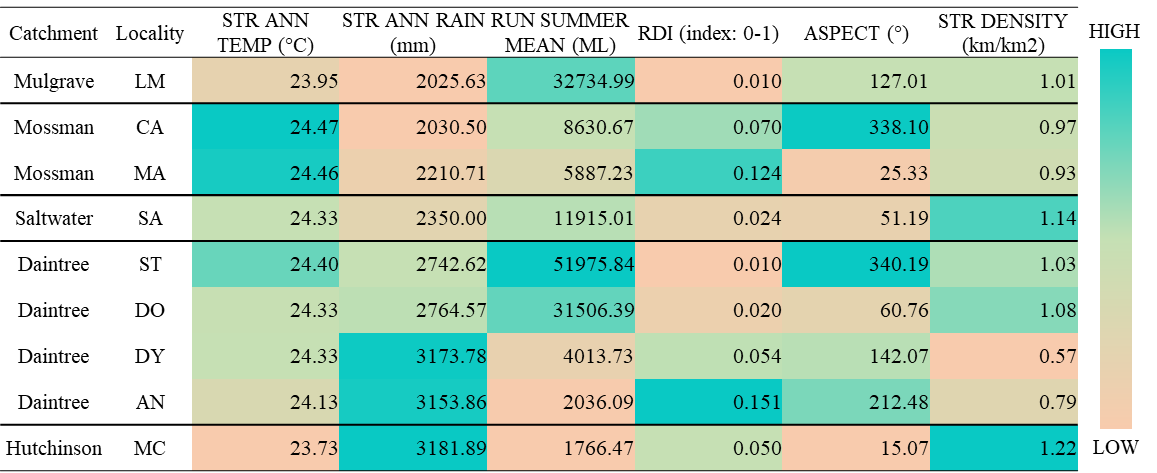


Figure S1g. Raw climate data for each sampling locality of *Melanotaenia splendida splendida*. Shading represents relative variation among sites specific to each variable. Locality abbreviations: LM = Little Mulgrave Creek, CA = Cassowary Creek, MA = Marrs Creek, SA = Saltwater Creek, ST = Stewart Creek, DO = Douglas Creek, DY = Doyle Creek, AN = Forest Creek, MC = McClean Creek.

*S1h. Genotype-environment associations*

The standard covariate model of _BAYPASS_ 2.2 (Gautier 2015) tests linear associations between each SNP and each of a set of given environmental variables. The auxiliary model, used here, extends upon this method by introducing a Bayesian auxiliary variable for each regression coefficient to indicate whether a SNP is associated with a given climatic variable. Posterior distributions are then evaluated to produce a Bayes Factor (BFmc) indicating strength of evidence for each association. The method implicitly corrects for multiple-testing effects, whereby an increase in the number of explanatory variables can increase the likelihood of false positives. First, we centred and scaled environmental variables in _R_ (*scale* function) to standardise comparisons relative to the total variation of each factor. We then ran the auxiliary model with default parameters to test associations between population-specific allele count data (14,540 SNPs) and the scaled environmental variables, while accounting for assumed population demographic structure (the scaled covariance matrix of population allele frequencies (Ω) resulting from the core model). Finally, the Bayes Factor estimates, and the underlying regression coefficients, were plotted in _R_ using the *plot* function.

For the RDAs, we began with the same 14,540 quality-filtered and unlinked SNPs previously converted to a GENIND object using the _R_ package _ADEGENET_. Genotypes were obtained from reference allele counts, then, missing data were replaced with the most common genotype for that locus. This is a conservative approach, in that it’s more likely to minimise than exaggerate differences between sampled populations. We also used the same set of centred and scaled environmental variables as for the _BAYPASS_ GEA analysis. To assess potential associations between genotype and environmental variables, we used the R package _VEGAN_ 2.5-6 (Oksanen et al. 2019) to perform the following functions. First, an initial global RDA was run using the six environmental variables as explanatory factors, and the 14,540 SNPs used as the multivariate response (*rda* function). The variance inflation factor (VIF; *vif.cca*) for the model was calculated to ensure that no instances of multicollinearity remained between explanatory variables, with a VIF ≤ 5 considered acceptable. Analyses of variance (ANOVAs; *anova.cca*) were used with 999 permutations to test the significance of the global model, as well as each of the constrained axes. The *ordistep* function was then used with backwards-stepwise selection to determine the best combination of explanatory variables and their relative contributions to the model. Only those found to have a significance of *p* ≤ 0.1 were used in subsequent partial RDAs.

While some GEA algorithms (e.g., the _BAYPASS_ auxiliary covariate model used above) implicitly account for the influence of neutral demographic variation, RDA methods require the partialing out of any potentially confounding explanatory factors by their inclusion in the model as conditional variables. Referred to as a partial RDA (pRDA), this method frequently incorporates a spatial conditional variable, either in the form of geographic coordinates or a measure of distance suited to the study system (e.g. waterway/stream distances, as in Brauer et al. (2016)). However, neither of these measures could be said to be an ideal representation of the likelihood of gene flow in the tropical rainbowfish study system, in which some geographically distant sampling locations are connected by the same river system, while others in proximity are separated by catchment boundaries. Moreover, neither of these methods can account for effects to connectivity due to strength, direction and perenniality of river flow, or the presence of artificial barriers such as dams and weirs. We therefore chose to account for distance using genetic measures, including fixation index (*F*_ST_; earlier obtained from analysis in _ADEGENET_) and covariance among population allele frequencies (Ω; earlier obtained from analysis in _BAYPASS_). For the sake of comparison, we also repeated the analysis conditioned with a geographic covariable devised to incorporate both waterway distances and separation among drainage systems. Pairwise distances among connected sites (within drainages) were characterised as shortest distances along flow paths of Surface Hydrology Lines (National) (Crossman and Li 2015) calculated in ARCMAP 10.3 (ESRI 2011). Waterway distances could not be calculated among sites without waterway connectivity. Therefore, since pairwise *F*_ST_ was approximately five times greater between drainages than within, we imputed an artificial pairwise distance variable for these unconnected sites five times greater than the average waterway distance.

For each conditioning variable, population values were expanded to individual-level matrices. We then performed principal coordinate analyses (PCoA) on the respective distances (*pcoa* function implemented in _R_ package _APE_ 5.3 (Paradis and Schliep 2019)), retaining only the significant PCo axes. Partial RDAs were then performed, controlling for each of the respective distance measures using explanatory variables identified as significant in the global model. As above, ANOVAs (999 permutations) were used to assess significance of the final RDA models, as well as the significance of the RDA axes within each model. Again, *ordistep* was used to assess the relative contribution of each of the explanatory variables. Finally, a list of candidate SNPs was established for each of the final RDAs (controlling for Fst, Ω, and waterway distances respectively) by identifying outliers ±3 standard deviations (two-tailed *p*-value = 0.0027) from the mean loading (i.e. the correlation between the observed score and the latent score) of each significant RDA axis, following recommendations of Forester et al. (2018).

*S1i. Geometric morphometric analysis*

Morphometric landmarks were chosen for homology, repeatability, and likelihood of ecological relevance. For instance, the majority represent intersections of fins or other skeletal structures, ensuring homology and providing a thorough representation of overall body shape and fin positioning. The only notable exceptions to homology are the front and rear margins of the maximum eye width (landmarks 3 and 4). However, these were included on the basis that the eye is an important sensory organ and might reflect ecologically relevant differences, and identification of these points are considered to be repeatable (Zelditch et al. 2012). The landmarks were also chosen to include those with ecological relevance in previous studies of rainbowfish morphology (McGuigan et al. 2003, McGuigan et al. 2005).


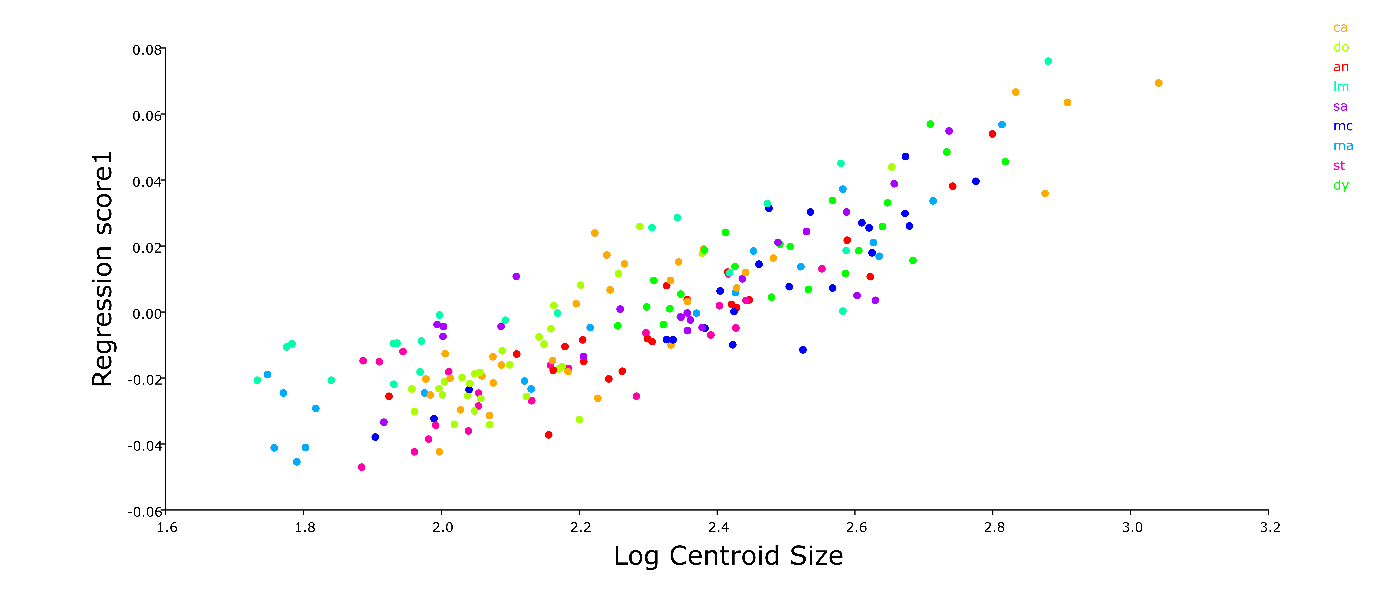


Figure S1i. Regression of individual Procrustes coordinates against log centroid size pooled by sampling site, for *Melanotaenia splendida splendida* sampled from the Wet Tropics of Queensland, with predicted 30.8% of shape variation explained by size (p=<0.0001). Locality codes: LM = Little Mulgrave Creek, CA = Cassowary Creek, MA = Marrs Creek, SA = Saltwater Creek, ST = Stewart Creek, DO = Douglas Creek, DY = Doyle Creek, AN = Forest Creek, MC = McClean Creek.

*S1j. Phenotype-environment associations*

To create the shape variable inputs, we processed the raw TPS files in _R_ using functions developed by Claude (2008). We used individual landmark configurations to build an array (*array*), which was once again subjected to a Procrustes superimposition (*pgpa*). From the resulting configurations, shape data was extracted using orthogonal projection (*orp*) to create a response matrix of individual Procrustes values. We then ran a PCA on the Procrustes matrix (*prcomp*) and used a broken stick model (*screeplot*) to determine which components of shape variation exceeded random expectations, to be retained for the RDA. From the Procrustes matrix, we also extracted values of individual centroid size, which were scaled (*scale*) and used to create a data frame for later inclusion as a covariable. Like genetic variation, body shape can also theoretically be influenced by adaptively neutral demographic structuring (Mitchell-Olds et al. 2007, Ho et al. 2017). As with the GEA analyses, we chose to account for neutral structure using fixation index (*F*_ST_; earlier obtained from analysis in _ADEGENET_) and covariance among population allele frequencies (Ω; earlier obtained from analysis in _BAYPASS_), and waterway distances (described in S1h). For each of these measures, we again expanded population values to individual-level matrices, before performing PCoAs (_R_ package _APE_, retaining only significant PCo axes. *N.B.* It should be noted that although *M. s. splendida* is sexually dimorphic, we did not control for sex in final model. Sex of rainbowfishes is usually determined by fin length and colour, both of which were observed to occur on a spectrum. This meant that confident identification was not possible for all individuals, and exclusion of ambiguous individuals would have limited analytical power due to a reduced sample size. However, for the majority which were able to be identified, sex ratios did not vary significantly between sampling sites (11:14 m:f, Chi-Square *p* value = 0.987) and should therefore be unlikely to bias either morphometric or PEA results.

We then used the _R_ package _VEGAN_ to run an initial global RDA using the six environmental variables as explanatory factors, and the four significant PCs as the multivariate response (*rda*). ANOVAs (*anova.cca*) were run with 999 permutations to test the significance of the global model, as well as each of the constrained axes. Backwards-stepwise selection (*ordistep*) was used to determine the best combination of explanatory variables and their relative contribution. Only those with *p* ≤ 0.1 were used in subsequent pRDAs. Two pRDAs were performed using explanatory environmental variables identified as significant in the global model, and the four significant PCs as the multivariate response (*rda*). They each controlled for the covariable of size, plus principal components of Ω, F*_ST_*, or waterway distance, respectively. We assessed significance of the final models, and the RDA axes contributing to each model, using ANOVA (*anova.cca*; 999 permutations). Finally, *ordistep* was used to assess the relative contribution of each of the explanatory variables.

*S1k. Genotype-phenotype-environment analysis*

In R, we ran a global RDA using the four significant principal components of individual Procrustes distances as explanatory variables, and 864 putative adaptive alleles (identified in the genotype-environment pRDA controlling for Ω) as the multivariate response; *N.B*., although we performed pRDAs controlling for both Ω and *F*_ST_ to confirm major patterns of environmental association, we chose, for simplicity, to use only adaptive candidates identified in the former analysis which has the advantage of model-based estimations of population covariance structure. The VIF (*vif.cca*) was calculated to ensure no multicollinearity between explanatory variables (VIF ≤ 5 considered acceptable). We used ANOVA (*anova.cca*, 999 permutations) to test significance of the global model, and the *ordistep* function to identify important explanatory variables. Those with significance of *p* ≤ 0.1 were used in the subsequent pRDA. This was performed in an identical manner, but with the introduction of size as a covariable. We again used ANOVA (*anova.cca*, 999 permutations) to test significance of the global model, as well as the significance of the RDA axes within each model. Backwards stepwise selection (*ordistep*) was used to assess the relative contribution of each explanatory shape PCo. A list of candidate SNPs was established for the partial RDA by identifying outliers ±2 standard deviations (two-tailed *p*-value = 0.0455) from the mean loading each significant RDA axis. This cut-off is less stringent than for the original GEA analysis (±3 std), allowing for the strong likelihood that body shape variation is polygenic in nature, and may be maintained by more subtle frequency shifts of individual alleles (Höllinger et al. 2019).

S2. Supplementary Results

*S2a. Genome-wide SNP data*

Table S2a. Total number of variant sites retained after each filtering step for mapped ddRADseq reads for the eastern rainbowfish *Melanotaenia splendida splendida*.

| Filtering Step | Number of SNPs |
| --- | --- |
| Raw catalogue | 9,827,129 |
| Genotyped in 80% of individuals, bi-allelic, minor allele frequency >0.03 | 62,277 |
| Indels removed | 56,745 |
| Read quality (quality/coverage depth >0.2) | 55,277 |
| Mapping quality score > 30 | 41,177 |
| Depth of coverage <mean+2SD | 39,964 |
| Missing data per locality <25% | 39,157 |
| Hardy–Weinberg equilibrium in >75% localities | 37,344 |
| Unlinked (>189 bp separation) | 14,540 |
| Putatively neutral (Bayescan) | 14,478 |


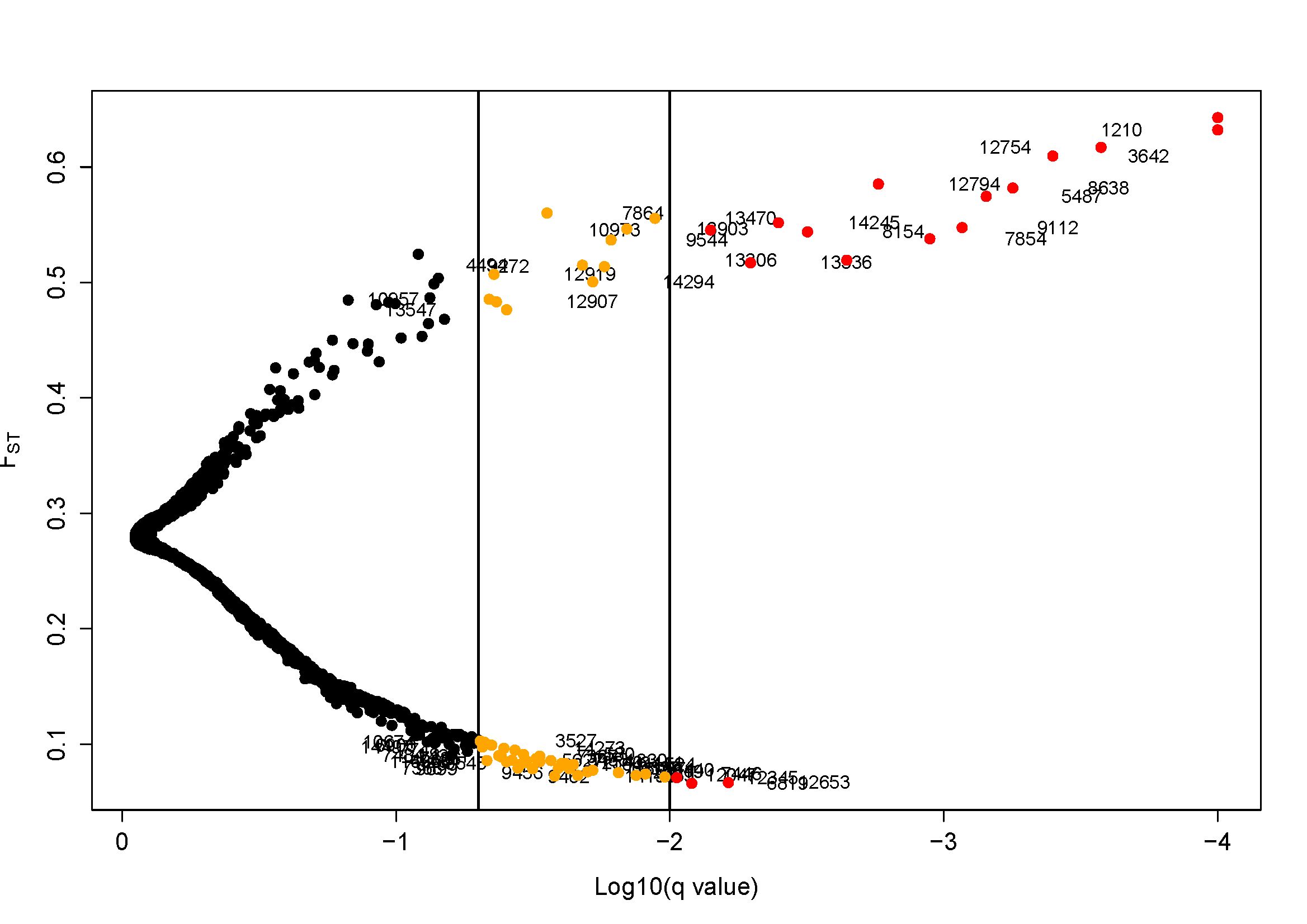
Figure S2a. Outlier analysis of 14,540 unliked SNPs for the eastern rainbowfish *Melanotaenia splendida splendida* using _BAYESCAN_ 2.1, showing the relationship between *F*_ST_ and log10-transformed q-values. For the neutral dataset, we removed 62 loci with greater differentiation than expected under neutrality (FDR = 0.05, first vertical line), retaining 14,478 putatively neutral SNPs.

*S2b. Pairwise F_ST_*

Table S2b. Pairwise F_ST_ among Melanotaenia splendida splendida from nine rainforest sampling sites based on 14,478 putatively neutral SNPs. Locality abbreviations: LM = Little Mulgrave Creek, CA = Cassowary Creek, MA = Marrs Creek, SA = Saltwater Creek, ST = Stewart Creek, DO = Douglas Creek, DY = Doyle Creek, AN = Forest Creek, MC = McClean Creek.

|  | LM | CA | MA | SA | ST | DO | DY | AN |
| --- | --- | --- | --- | --- | --- | --- | --- | --- |
| CA | 0.127 |  |  |  |  |  |  |  |
| MA | 0.126 | 0.026 |  |  |  |  |  |  |
| SA | 0.158 | 0.075 | 0.071 |  |  |  |  |  |
| ST | 0.105 | 0.087 | 0.084 | 0.111 |  |  |  |  |
| DO | 0.108 | 0.088 | 0.086 | 0.113 | 0.017 |  |  |  |
| DY | 0.119 | 0.099 | 0.097 | 0.124 | 0.029 | 0.028 |  |  |
| AN | 0.109 | 0.090 | 0.089 | 0.115 | 0.021 | 0.019 | 0.028 |  |
| MC | 0.208 | 0.174 | 0.174 | 0.202 | 0.127 | 0.130 | 0.141 | 0.132 |

*S2c. Discriminant Analysis of Principal Components*


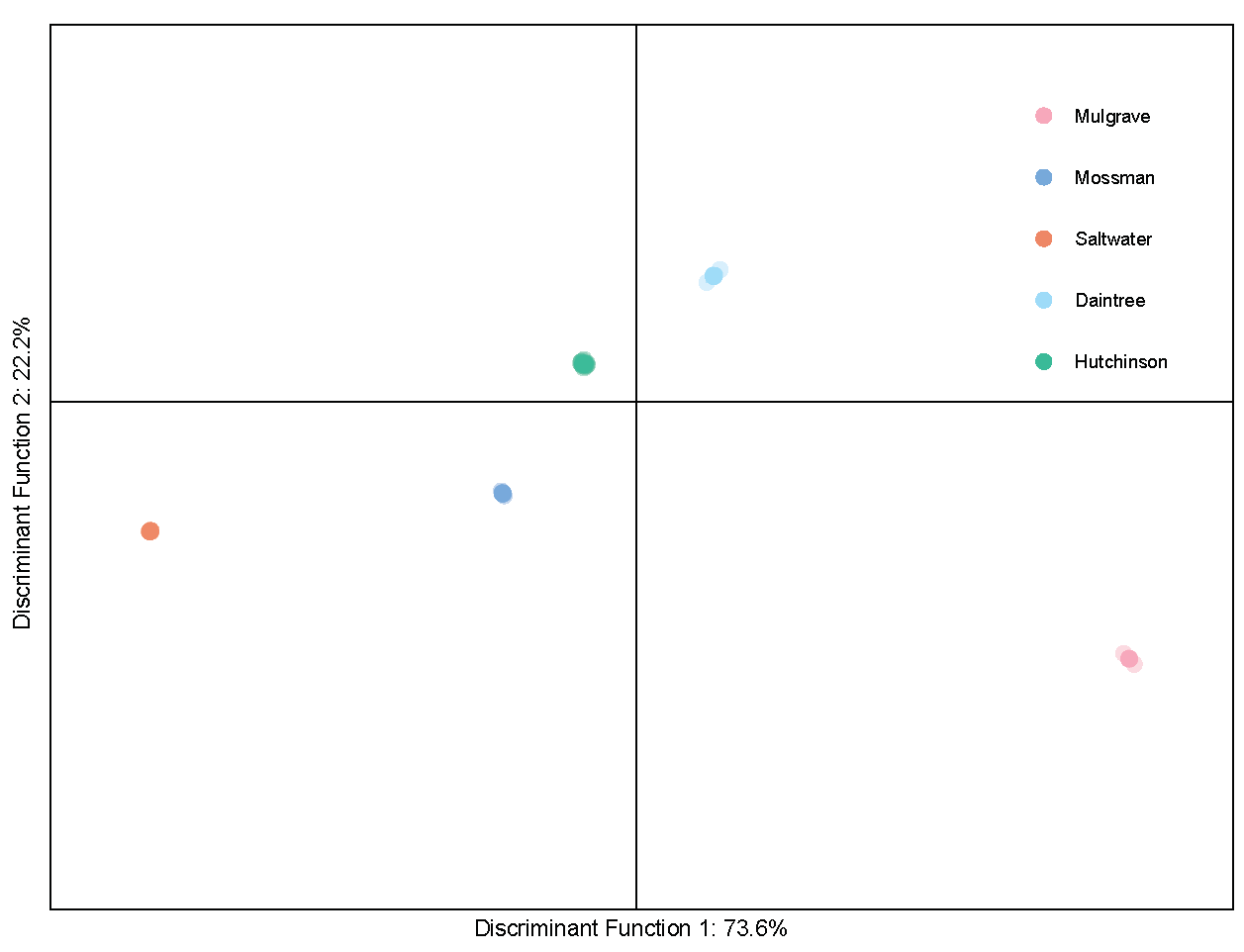


Figure S2c. Discriminant analysis of principal components of putatively neutral genetic variation (14,478 SNPs) for the eastern rainbowfish (*Melanotaenia splendida splendida*) individuals sampled from nine localities among five drainage systems in the Wet Tropics of Queensland. Colours correspond simultaneously to drainage and the most likely group membership inferred by the model (K = 5).

*S2d. Neighbour-joining Tree*

**

Figure S2d. Unrooted neighbour-joining tree for individual genetic distances (TN93) based on 14,478 putatively neutral loci for the rainbowfish *Melanotaenia splendida splendida* in the Wet Tropics of Queensland. Colours loosely encircle individuals by drainage system of origin (Mulgrave, Mossman, Saltwater, Daintree, Hutchinson).

*S2e Migration Rates*


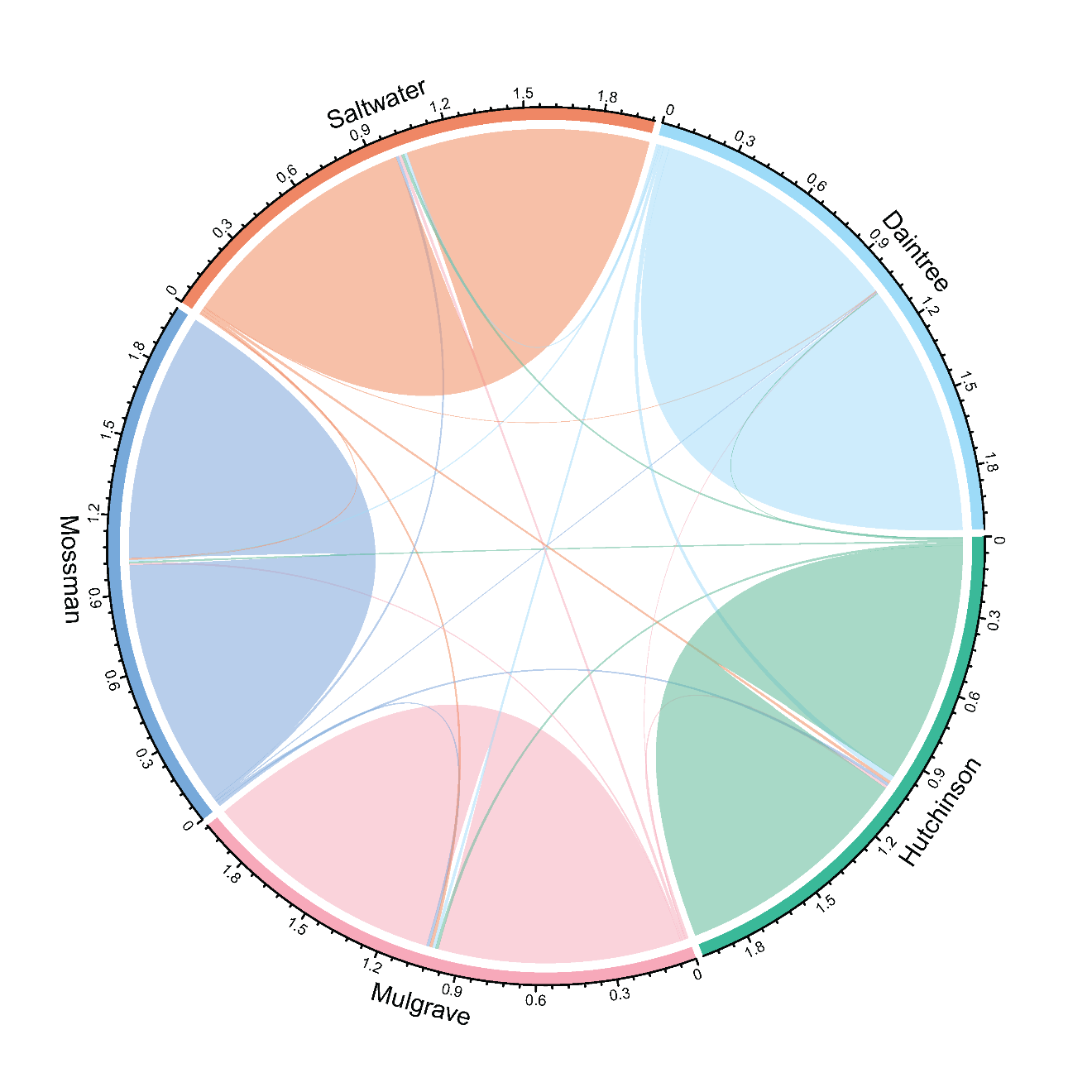


Figure S2e Average estimates of contemporary migration rates (m) among populations of *Melanotaenia splendida splendida* based on 14,478 putatively neutral SNPs. Migration estimates are represented by curve thickness (see Table S2e for numerical values). Colours represent catchment of origin.

Table S2e. Average estimates of contemporary migration rates (m) into each population of *Melanotaenia splendida splendida*. The recipient populations are listed in the rows, while the origins of the migrants are listed in the columns. Standard deviations for all distributions were <0.05.

|  | Mulgrave | Mossman | Saltwater | Daintree | Hutchinson |
| --- | --- | --- | --- | --- | --- |
| Mulgrave | 0.9523 | 0.0071 | 0.0116 | 0.0033 | 0.0111 |
| Mossman | 0.0120 | 0.9720 | 0.0115 | 0.0034 | 0.0109 |
| Saltwater | 0.0120 | 0.0069 | 0.9540 | 0.0033 | 0.0109 |
| Daintree | 0.0119 | 0.0071 | 0.0116 | 0.9865 | 0.0223 |
| Hutchinson | 0.0119 | 0.0070 | 0.0113 | 0.0035 | 0.9448 |

*S2e. Partial RDA model: variable contributions*

Figure S2e. Percentage stacked column graph representing variance partitioning of pRDA response variables (genomic variation or morphological variation of *Melanotaenia splendida splendida*) among environmental explanatory variables (Table 2, main text) and neutral covariables (allelic covariance (Ω); *F*_ST_ distances (*F*ST), waterway distances (River dist)). Colours correspond to proportion of variation best explained by environmental variables = “Environment”; by neutral variables = “Neutral”; by environmental or neutral variables equally = “Overlapping” .

*S2f. Global redundancy analysis of genotype-environment associations*


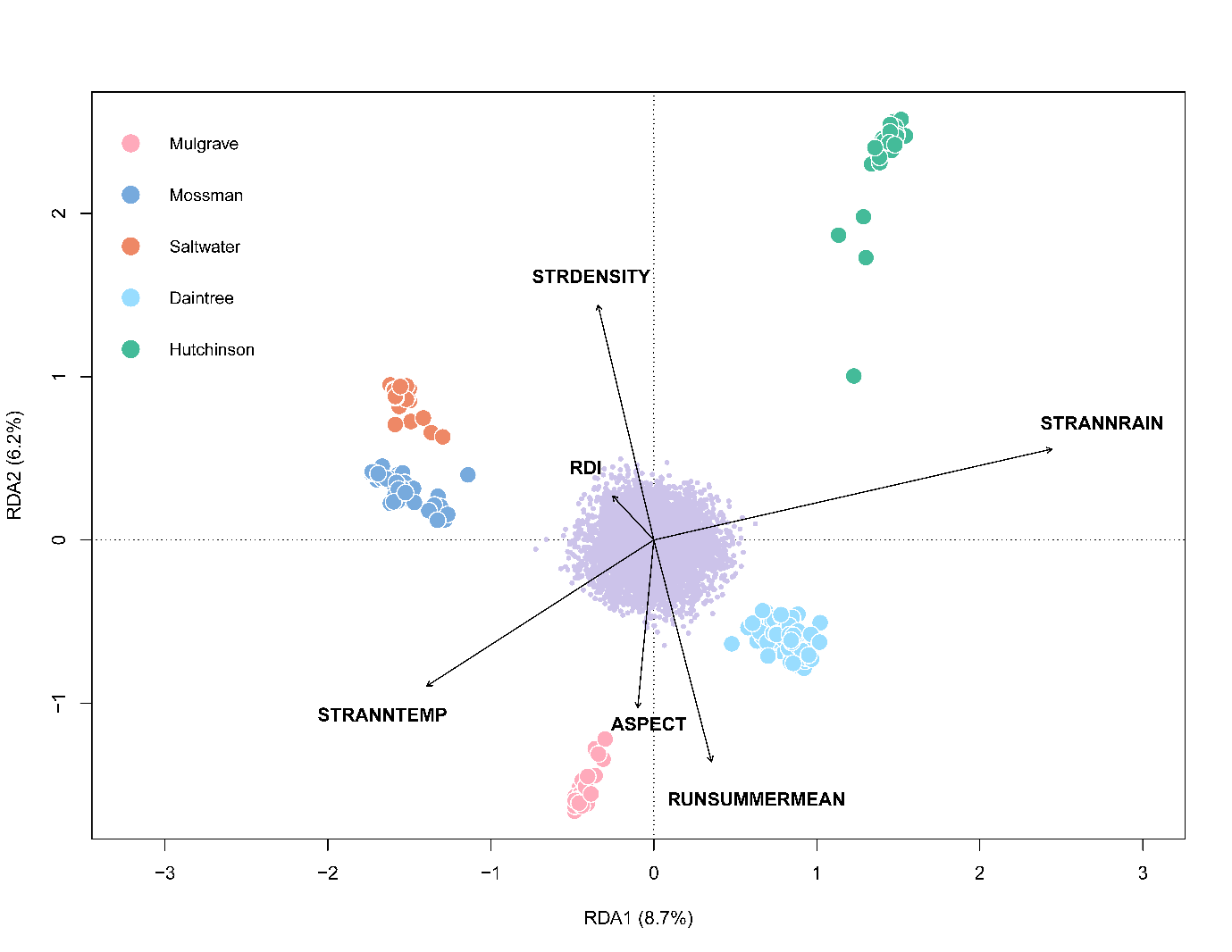


Figure S2f. Ordination plot summarising the first two axes of a global redundancy analysis for genomic variation (14,540 SNPs) of *Melanotaenia splendida splendida* individuals as explained by six significantly associated environmental variables (p = <0.001). Large points represent individual-level responses, and are coloured by drainage system of origin. Small purple points represent SNP-level responses. Vectors represent the magnitude and direction of relationships with explanatory variables.

*S2g. GEA candidate loci identified by partial redundancy analysis, controlling for allelic covariance*


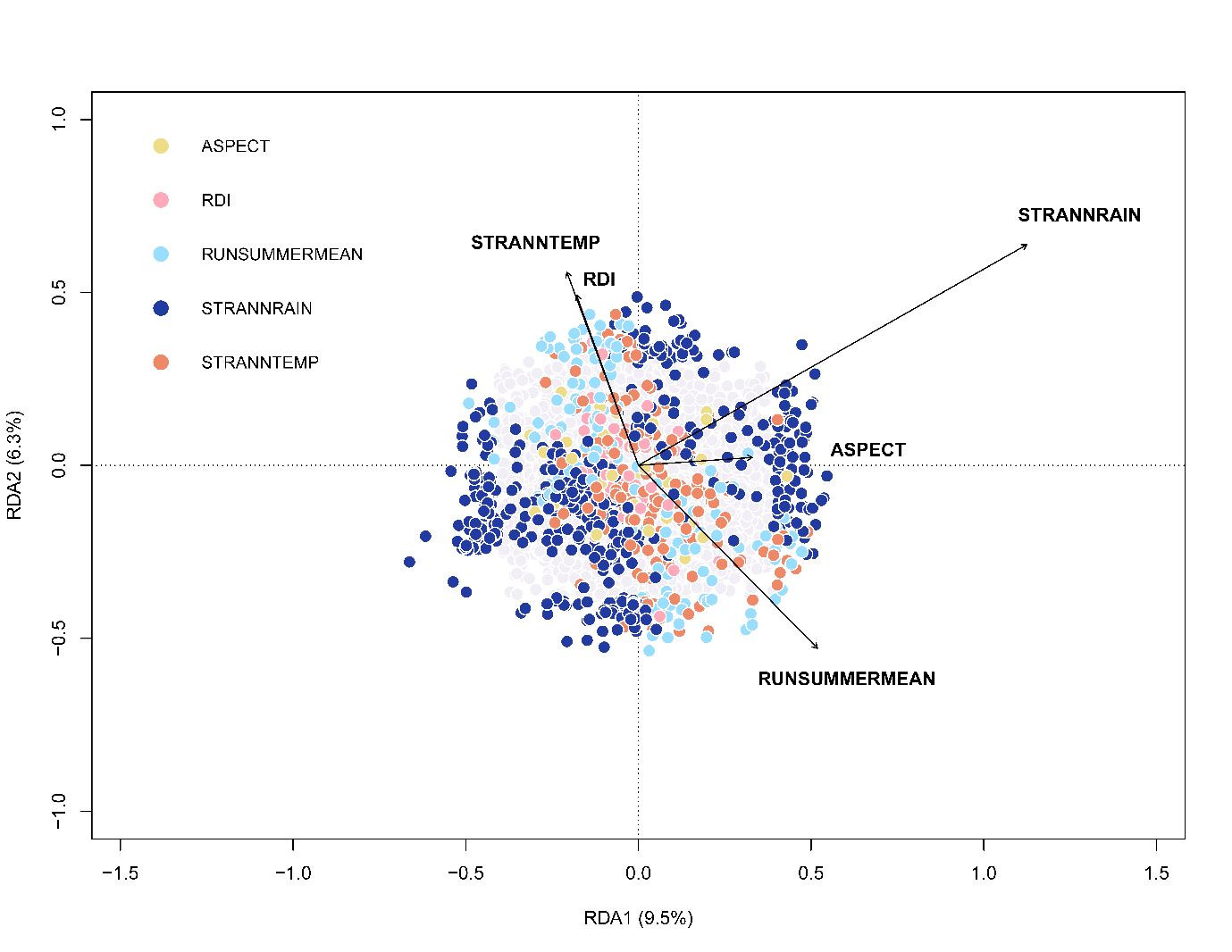


Figure S2g. Partial redundancy analysis (pRDA) showing variation of 14540 SNPs from *Melanotaenia splendida splendida* rainforest individuals in relation to five environmental predictor variables, after controlling for Ω (allelic covariance) among sampling localities. The 864 SNPs represented by coloured points were strongly and significantly associated with at least one environmental predictor (p ≤ 0.0027; colour key indicates best predictor variable), while SNPs represented by light grey points were unassociated. Vectors represent the magnitude and direction of relationships with explanatory variables.

*S2h. Partial redundancy analysis of genotype-environment associations, controlling for F_ST_*


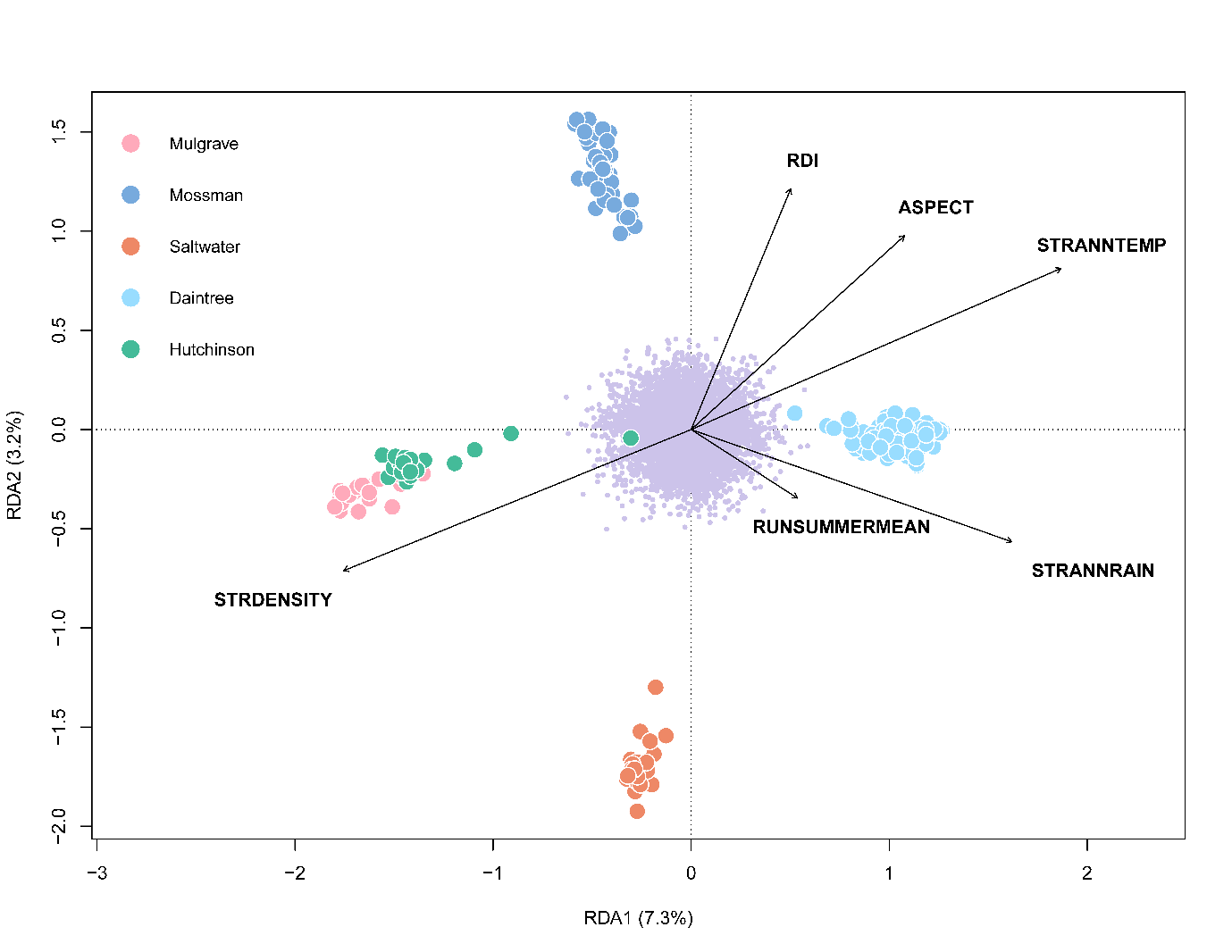


Figure S2h. Ordination plot summarising the first two axes of a partial redundancy analysis for genomic variation (14,540 SNPs) of *Melanotaenia splendida splendida* individuals as explained by six significantly associated environmental variables, (p = <0.001) after controlling for pairwise *F*_ST_ among sampling localities. Large points represent individual-level responses, and are coloured by drainage system of origin. Small purple points represent SNP-level responses. Vectors represent the magnitude and direction of relationships with explanatory variables.

*S2i. Partial redundancy analysis of genotype-environment associations, controlling for geographic (waterway) distance*


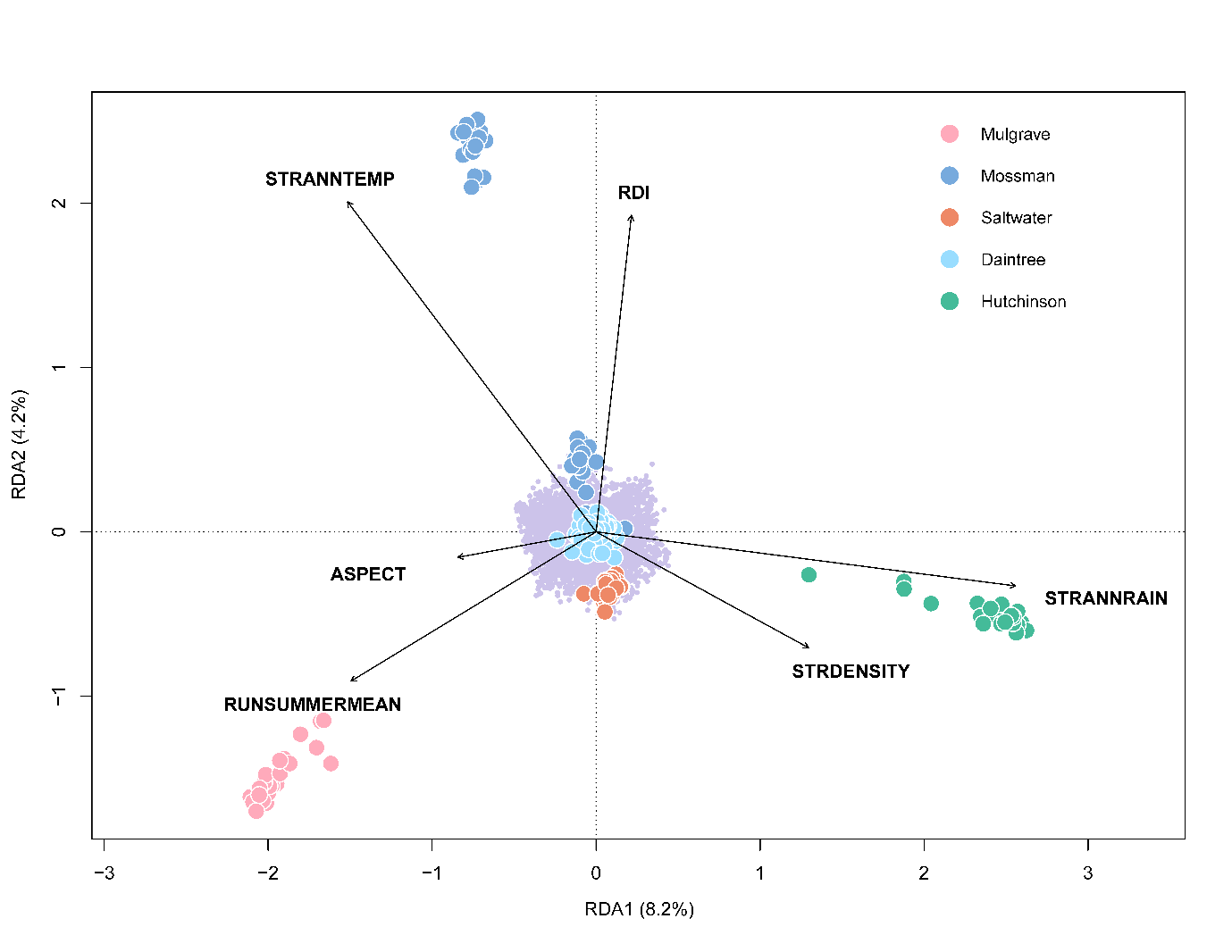


Figure S2i. Ordination plot summarising the first two axes of a partial redundancy analysis for morphometric variation (18 landmarks) of *Melanotaenia splendida splendida* individuals as explained by six significantly associated environmental variables, (p = <0.05) after controlling for waterway distances among sampling localities. Large points represent individual-level responses, and are coloured by drainage system of origin. Small purple points represent SNP-level responses. Vectors represent the magnitude and direction of relationships with explanatory variables.


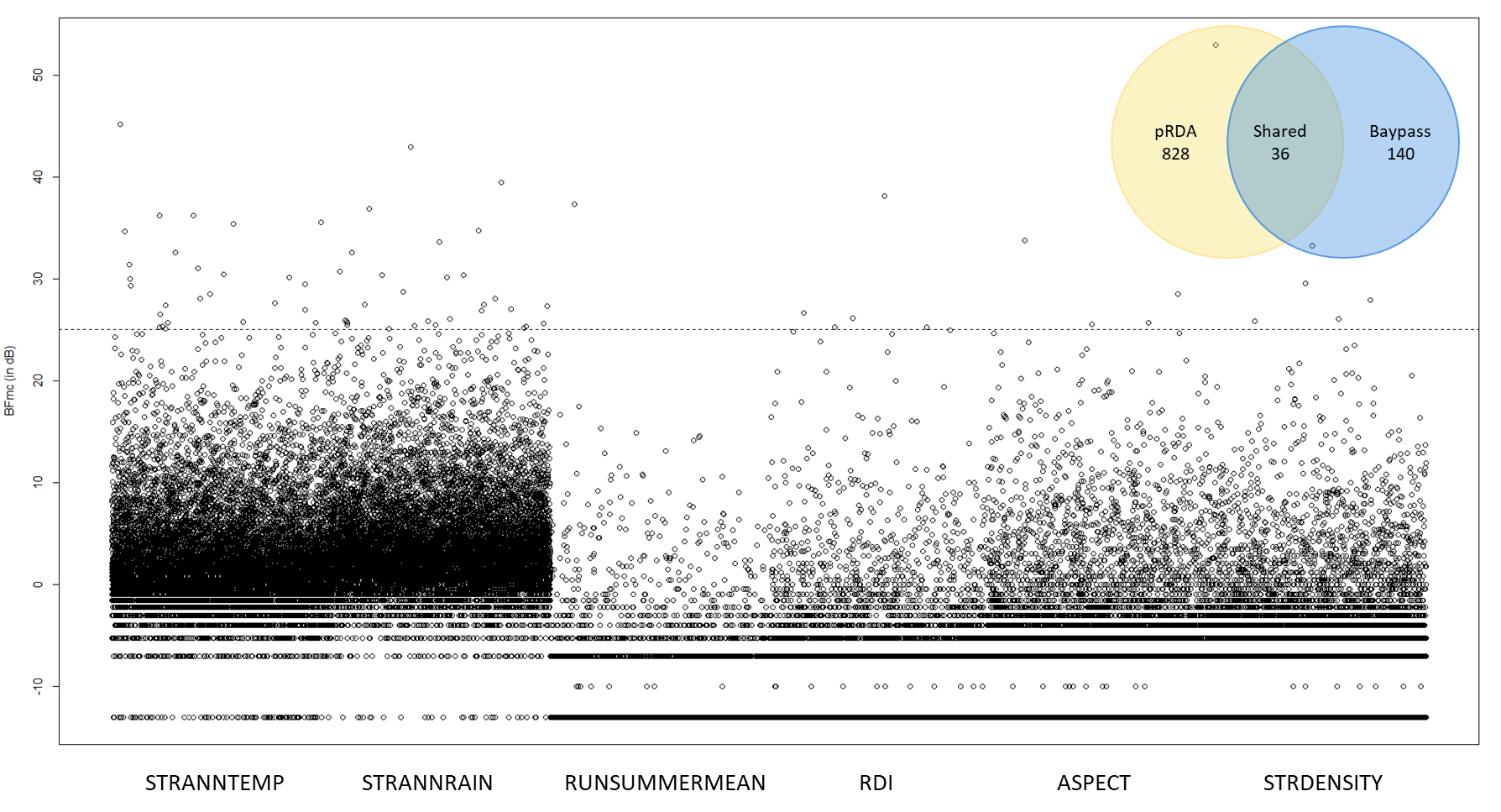
*S2j. Genotype environment associations using _BAYPASS_ auxiliary covariate model*

Figure S2j. Climatic association of 14,540 SNPs from *Melanotaenia splendida splendida* across nine rainforest sampling sites against six independent environmental variables using _BAYPASS_ auxiliary covariate model. Dashed line indicates Bayes Factor cutoff of 21.46 dB (99.8% probability), above which 176 loci were identified as candidates for climatic adaptation. Inset: Venn diagram indicates the number of unique and shared candidates among GEA analyses using pRDA and Baypass approaches.

*S2k. Morphometric variation among localities*


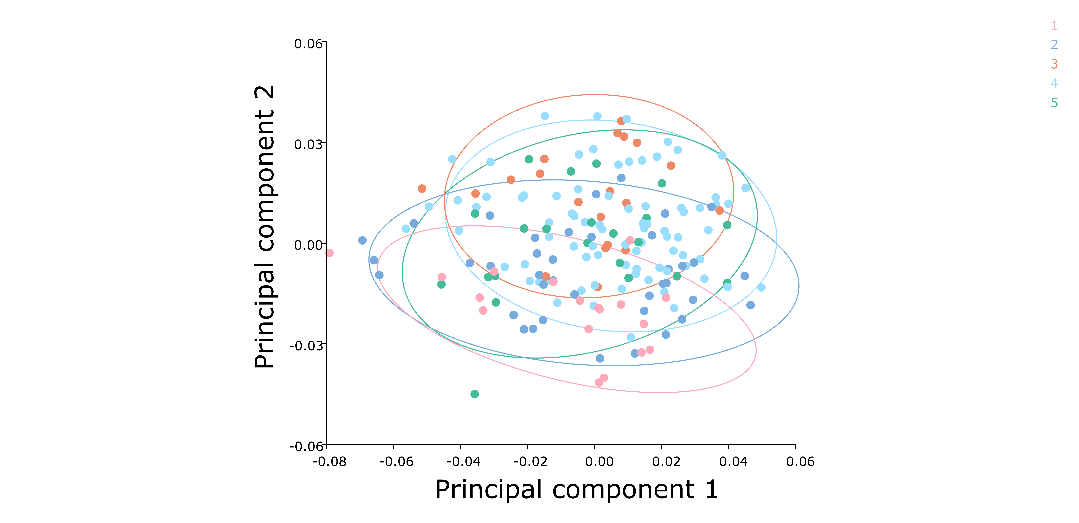

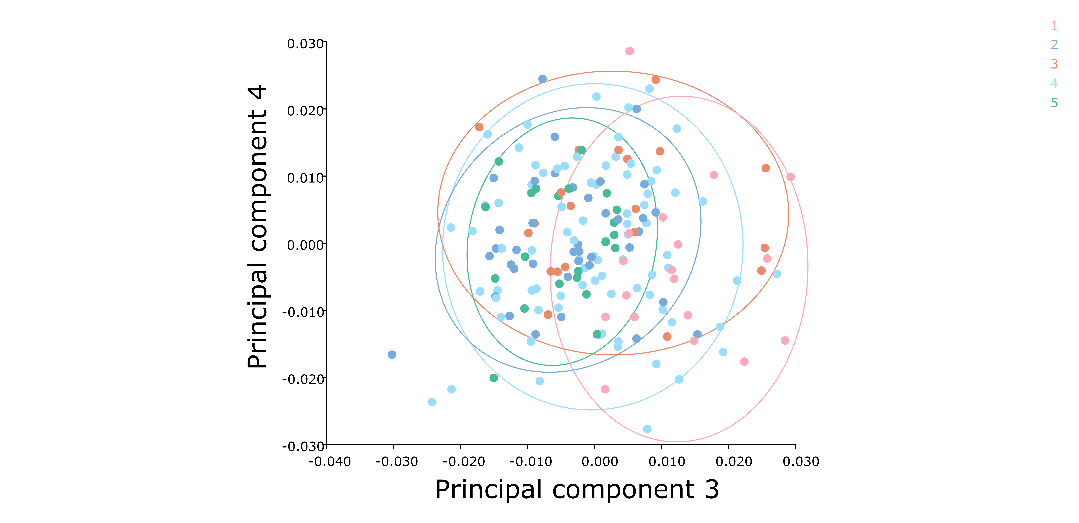


Figure S2k. Significant principal components of body shape variation for Melanotaenia splendida splendida individuals sampled across the Wet Tropics of Queensland. PCA scatterplots show relative variation among individuals, with colours and equal frequency ellipses (90% probability) show for drainage system of origin (Mulgrave, Mossman, Saltwater, Daintree, Hutchinson).

*S2l. Canonical variate analysis of body shape variation*


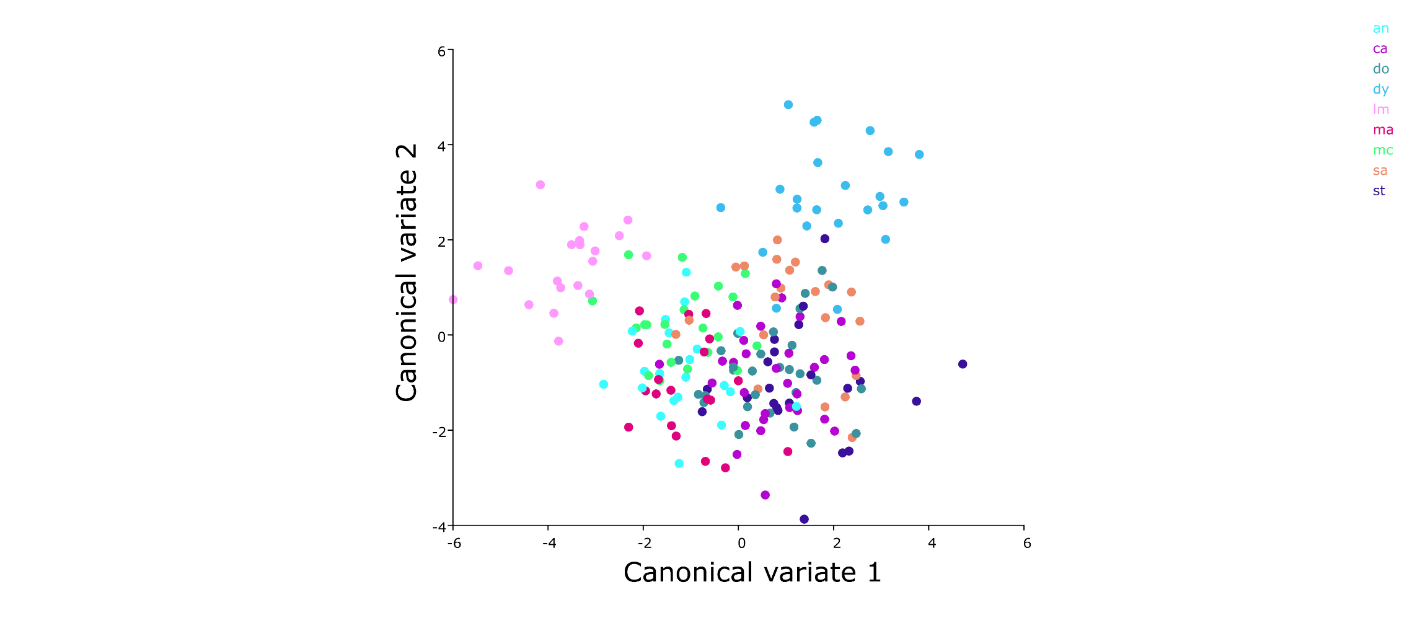


LM

CA

MA

SA

ST

DO

DY

AN

MC

Figure S2l. Canonical variate analysis of body shape variation of *Melanotaenia splendida splendida* among nine rainforest sampling sites. Locality codes: LM = Little Mulgrave Creek, CA = Cassowary Creek, MA = Marrs Creek, SA = Saltwater Creek, ST = Stewart Creek, DO = Douglas Creek, DY = Doyle Creek, AN = Forest Creek, MC = McClean Creek.

Table S2l. Procrustes distances among sampling sites and among drainage systems, based on canonical variate analysis of body shape of *Melanotaenia splendida splendida*. Locality codes: LM = Little Mulgrave Creek, CA = Cassowary Creek, MA = Marrs Creek, SA = Saltwater Creek, ST = Stewart Creek, DO = Douglas Creek, DY = Doyle Creek, AN = Forest Creek, MC = McClean Creek. P-values from 10000 permutations: *** = p<0.01, ** = p<0.05, * = p<0.10.

|  | BY SAMPLING SITE | | | | | | | |
| --- | --- | --- | --- | --- | --- | --- | --- | --- |
|  | AN | CA | DO | DY | LM | MA | MC | SA |
| CA | 0.014*** |  |  |  |  |  |  |  |
| DO | 0.015*** | 0.016*** |  |  |  |  |  |  |
| DY | 0.026*** | 0.029*** | 0.024*** |  |  |  |  |  |
| LM | 0.033*** | 0.030*** | 0.035*** | 0.041*** |  |  |  |  |
| MA | 0.015*** | 0.015*** | 0.015*** | 0.030*** | 0.025*** |  |  |  |
| MC | 0.008 | 0.016*** | 0.014** | 0.024*** | 0.034*** | 0.016*** |  |  |
| SA | 0.024*** | 0.024*** | 0.016*** | 0.017*** | 0.040*** | 0.025*** | 0.021*** |  |
| ST | 0.018*** | 0.023*** | 0.013** | 0.023*** | 0.043*** | 0.024*** | 0.015** | 0.015** |
|  | BY DRAINAGE SYSTEM | | | | | | | |
|  | Mulgrave | Mossman | Saltwater | Daintree |  |  |  |  |
| Mossman | 0.027*** |  |  |  |  |  |  |  |
| Saltwater | 0.040*** | 0.023*** |  |  |  |  |  |  |
| Daintree | 0.036*** | 0.016*** | 0.013*** |  |  |  |  |  |
| Hutchinson | 0.034*** | 0.014*** | 0.021*** | 0.012** |  |  |  |  |

*S2m. Global redundancy analysis of phenotype-environment associations*


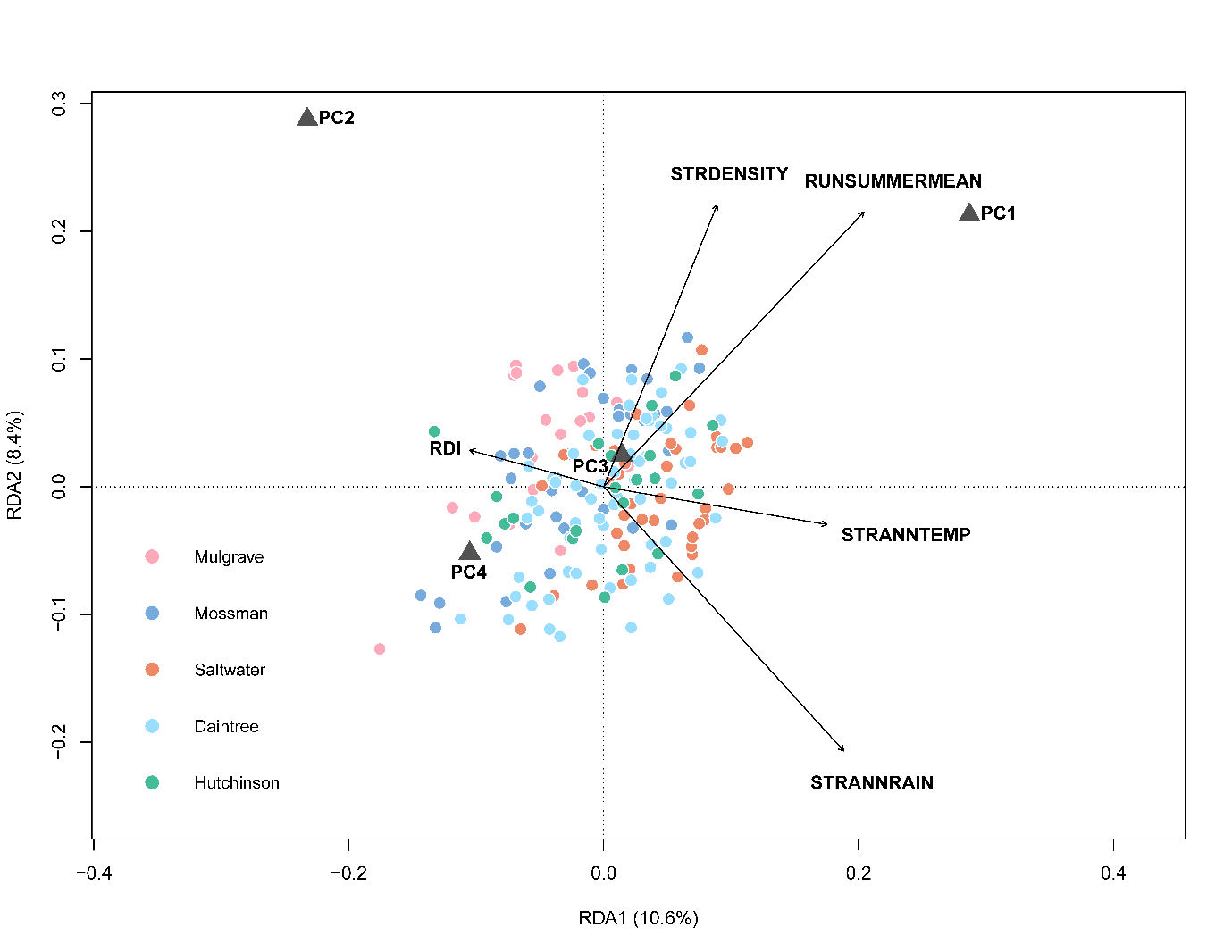
Figure S2m. Ordination plot summarising the first two axes of a global redundancy analysis for body shape variation (18 landmarks) of *Melanotaenia splendida splendida* individuals as explained by four significantly associated environmental variables (p = <0.001). Points represent individual-level responses, and are coloured by drainage system of origin. Vectors represent the magnitude and direction of relationships with explanatory variables.

*S2n. Partial redundancy analysis of phenotype-environment associations, controlling for F*_ST_ *and body size*


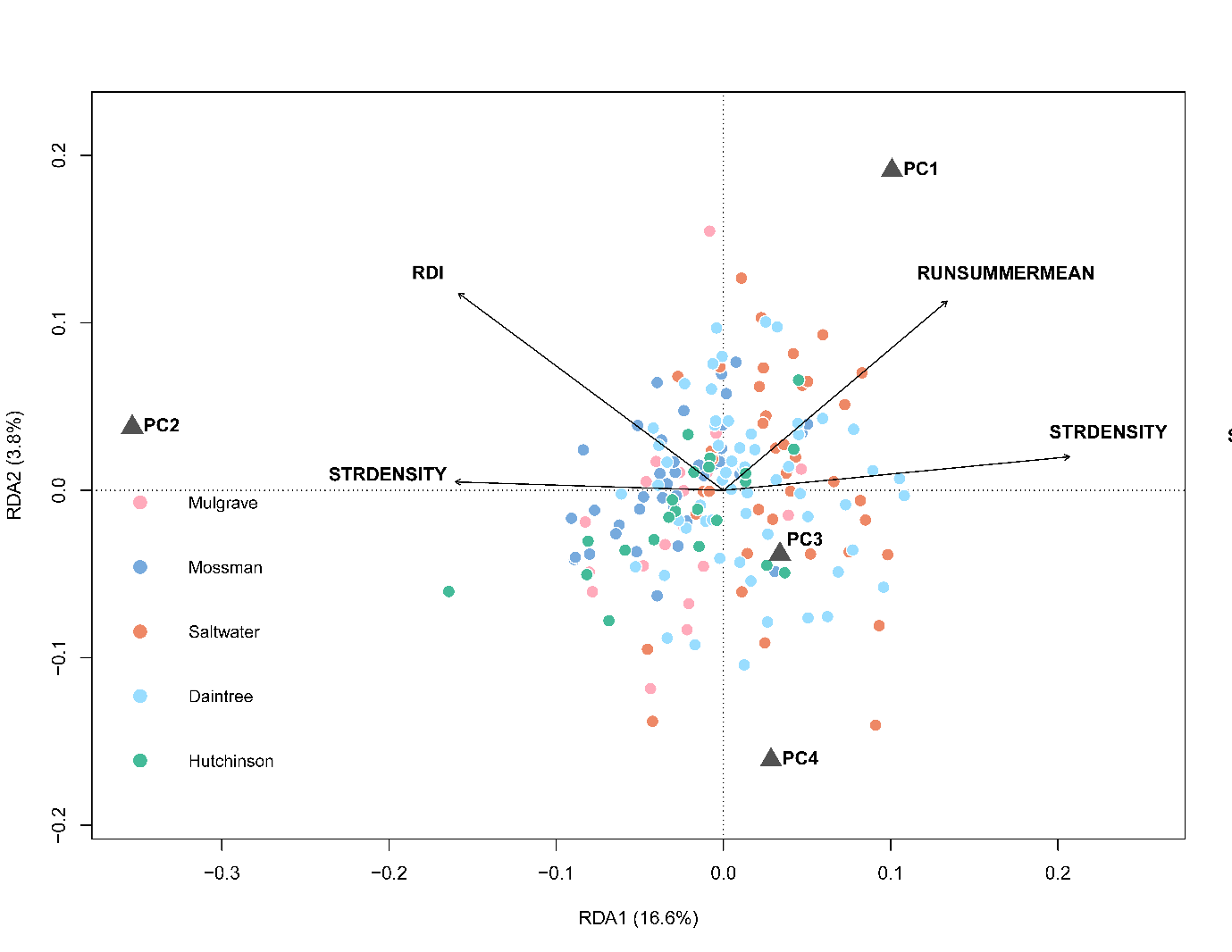


Figure S2n. Ordination plot summarising the first two axes of a partial redundancy analysis for body shape variation (18 landmarks) of *Melanotaenia splendida splendida* individuals as explained by four significantly associated environmental variables (p = <0.001) ), after controlling for individual body size and pairwise *F*_ST_ among sampling localities. Points represent individual-level responses, and are coloured by drainage system of origin. Vectors represent the magnitude and direction of relationships with explanatory variables.

*S2o. Partial redundancy analysis of phenotype-environment associations, controlling for geographic distance and body size*


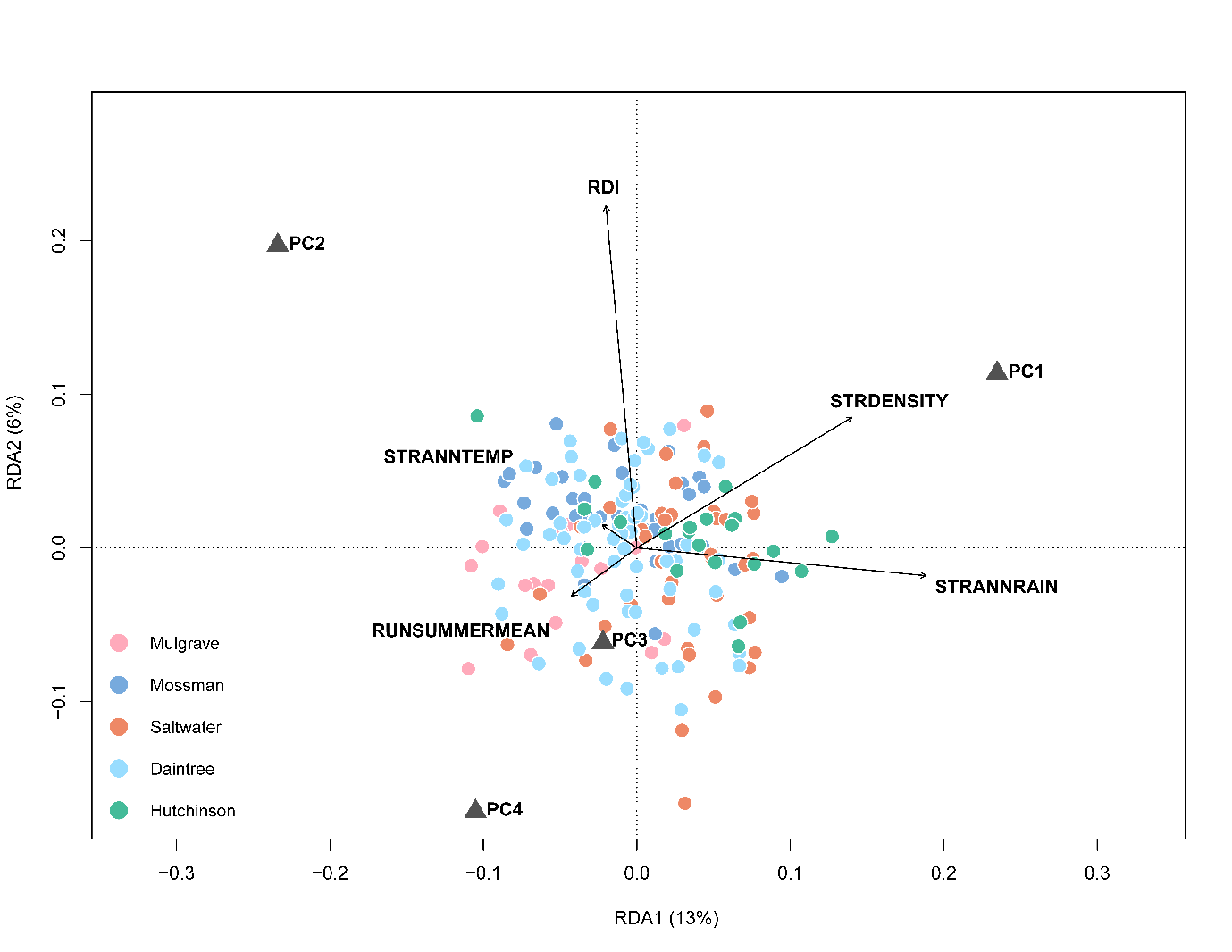
Figure S2o. Ordination plot summarising the first two axes of a partial redundancy analysis for body shape variation (18 landmarks) of *Melanotaenia splendida splendida* individuals as explained by four significantly associated environmental variables (p = <0.001) ), after controlling for individual body size and river distances among sampling localities. Points represent individual-level responses, and are coloured by drainage system of origin. Vectors represent the magnitude and direction of relationships with explanatory variables.

### S2p. GxPxE candidate loci identified by partial redundancy analysis


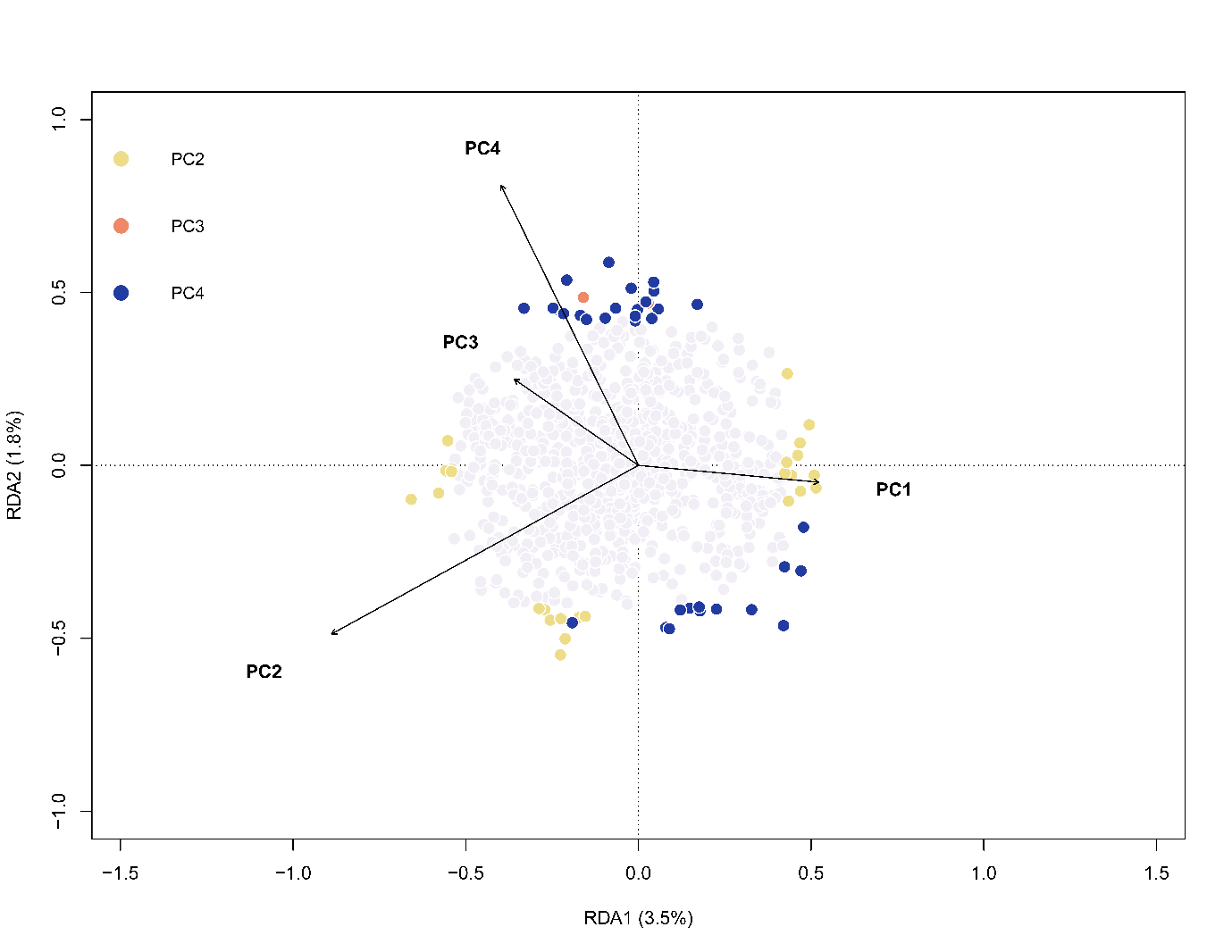


Figure S2p. Partial redundancy analysis (RDA) showing variation of 864 SNPs from *Melanotaenia splendida splendida* rainforest individuals in relation to four principal components (PCs) of body shape, after controlling for body size. The 61 SNPs represented by coloured points were significantly associated with at least one body shape PC (p ≤ 0.0455; colour key indicates best predictor variable), while SNPs represented by light grey points were not. Vectors represent the magnitude and direction of relationships with explanatory variables.

*S2q. Annotation of GEA and GxPxE candidate loci*

Table S2q. Functional annotation and gene ontology terms associated with 128 of 864 candidate loci for ecological adaptation in rainforest populations of *Melanotaenia splendida splendida* (e-value <= 1e-3). Candidates also identified by genotype-phenotype-environement associations are highlighted in grey.

| SNP ID | Scaffold no. & pos. | Gene | Protein | Cellular Component | Molecular Function | Biological Process |
| --- | --- | --- | --- | --- | --- | --- |
| 96 | 46:408496-409496 | Ppp1r16b | Protein phosphatase 1 regulatory inhibitor subunit 16B | cell projection, nuclear speck, nucleus, perinuclear region of cytoplasm, plasma membrane, | myosin phosphatase regulator activity, protein phosphatase 1 binding, protein phosphatase regulator activity, | regulation of sprouting angiogenesis |
| 137 | 43:212452-213452 | pcnx1 | Pecanex-like protein 1 | integral component of membrane, |  |  |
| 342 | 86:136769-137769 | TMF1 | TATA element modulatory factor | cytosol, endoplasmic reticulum, Golgi apparatus, Golgi membrane, nucleus, | androgen receptor binding, DNA binding, nuclear receptor coactivator activity, | spermatid nucleus differentiation |
| 371 | 39:1562871-1563871 | PRLHR | Prolactin-releasing peptide receptor | cilium, integral component of plasma membrane, plasma membrane, | G protein-coupled receptor activity, neuropeptide receptor activity, neuropeptide Y receptor activity, | hormone metabolic process |
| 382 | 39:2027055-2028055 | Entpd4 | Ectonucleoside triphosphate diphosphohydrolase 4 | autophagosome membrane, cytoplasmic vesicle, Golgi apparatus, Golgi membrane, integral component of membrane, lysosomal membrane, membrane, | CTPase activity, cytidine-diphosphatase activity, guanosine-diphosphatase activity, nucleoside-diphosphatase activity, nucleoside-triphosphatase activity, uridine-diphosphatase activity, | UDP catabolic process |
| 506 | 104:155871-156871 | STK11IP | Serine/threonine-protein kinase 11-interacting protein | azurophil granule lumen, cytoplasm, extracellular region, intracellular membrane-bounded organelle, lysosomal membrane, | protein kinase binding, | protein localization |
| 590 | 107:500411-501411 | Snx18 | Sorting nexin-18 | cytoplasmic vesicle, extracellular exosome, extrinsic component of cytoplasmic side of plasma membrane, growth cone, neuronal cell body, recycling endosome membrane, | phosphatidylinositol binding, phosphatidylinositol-4,5-bisphosphate binding, | protein transport |
| 640 | 59:85320-86320 | ctr9 | RNA polymerase-associated protein CTR9 homolog | Cdc73/Paf1 complex, euchromatin, nuclear speck, |  | transcription elongation from RNA polymerase II promoter |
| 781 | 108:327556-328556 | Rab12 | Ras-related protein Rab-12 | autophagosome, endosome, Golgi apparatus, Golgi membrane, insulin-responsive compartment, lysosomal membrane, lysosome, phagocytic vesicle, plasma membrane, recycling endosome, recycling endosome membrane, secretory granule, synaptic vesicle, | GDP binding, GTP binding, GTPase activity, | vesicle docking involved in exocytosis |
| 790 | 108:670912-671912 | miga1 | Mitoguardin 1 | integral component of plasma membrane, mitochondrial outer membrane, | protein heterodimerization activity, protein homodimerization activity, | mitochondrial fusion |
| 911 | 92:4269-5269 | Pc | Pyruvate carboxylase, mitochondrial | cytoplasm, mitochondrial matrix, mitochondrion, | ATP binding, biotin binding, carboxylic acid binding, identical protein binding, metal ion binding, pyruvate carboxylase activity, | viral RNA genome packaging |
| 953 | 140:77387-78387 | Map3k11 | Mitogen-activated protein kinase kinase kinase 11 | centrosome, cytoplasm, microtubule, | ATP binding, identical protein binding, JUN kinase kinase kinase activity, MAP kinase kinase kinase activity, mitogen-activated protein kinase kinase binding, mitogen-activated protein kinase kinase kinase binding, protein homodimerization activity, protein kinase activity, protein serine kinase activity, protein serine/threonine kinase activity, protein serine/threonine/tyrosine kinase activity, small GTPase binding, | protein phosphorylation |
| 1048 | 122:619839-620839 | GPAA1 | Glycosylphosphatidylinositol anchor attachment 1 protein | centrosome, cytosol, endoplasmic reticulum, endoplasmic reticulum membrane, GPI-anchor transamidase complex, membrane, mitochondrion, | tubulin binding, | proteolysis |
| 1149 | 171:8393-9393 | MYH7 | Myosin-7 | myofibril, myosin complex, myosin filament, sarcomere, | actin filament binding, ATP binding, calmodulin binding, cytoskeletal motor activity, |  |
| 1153 | 171:21896-22896 | Pak6 | Serine/threonine-protein kinase PAK 6 | cell junction, cytoplasm, fibrillar center, nucleoplasm, | ATP binding, protein serine kinase activity, protein serine/threonine kinase activity, protein serine/threonine/tyrosine kinase activity, | regulation of MAPK cascade |
| 1161 | 171:180617-181617 | CKB | Creatine kinase B-type | cytosol, extracellular membrane-bounded organelle, extracellular space, mitochondrion, | ATP binding, creatine kinase activity, kinase activity, ubiquitin protein ligase binding, | phosphocreatine biosynthetic process |
| 1206 | 123:673126-674126 | Safb | Scaffold attachment factor B1 | midbody, nucleoplasm, nucleus, | chromatin binding, RNA binding, RNA polymerase II cis-regulatory region sequence-specific DNA binding, sequence-specific DNA binding, | regulation of transcription by RNA polymerase II |
| 1239 | 191:237350-238350 | Atp1a3 | Sodium/potassium-transporting ATPase subunit alpha-3 | axon, calyx of Held, cytoplasm, dendritic spine head, dendritic spine neck, endoplasmic reticulum, Golgi apparatus, integral component of presynaptic membrane, membrane, myelin sheath, neuron to neuron synapse, neuronal cell body, nucleus, organelle membrane, photoreceptor inner segment, plasma membrane, postsynapse, sarcolemma, sodium:potassium-exchanging ATPase complex, synapse, | amyloid-beta binding, ATP binding, ATP hydrolysis activity, chaperone binding, D1 dopamine receptor binding, heparan sulfate proteoglycan binding, ion antiporter activity involved in regulation of presynaptic membrane potential, metal ion binding, P-type sodium:potassium-exchanging transporter activity, P-type sodium:potassium-exchanging transporter activity involved in regulation of cardiac muscle cell membrane potential, | visual learning |
| 1258 | 186:37803-38803 | Etfb | Electron transfer flavoprotein subunit beta | electron transfer flavoprotein complex, mitochondrial matrix, | electron transfer activity, nucleotide binding, | respiratory electron transport chain |
| 1291 | 190:269671-270671 | Ksr2 | Kinase suppressor of Ras 2 | cytoplasm, cytosol, plasma membrane, | ATP binding, MAP-kinase scaffold activity, metal ion binding, mitogen-activated protein kinase kinase binding, protein kinase activity, protein serine kinase activity, protein serine/threonine kinase activity, protein serine/threonine/tyrosine kinase activity, | signal transduction |
| 1597 | 183:417488-418488 | NOX4 | NADPH oxidase 4 | endoplasmic reticulum membrane, focal adhesion, integral component of membrane, plasma membrane, | oxidoreductase activity, |  |
| 1607 | 183:974116-975116 | HIP1 | Huntingtin-interacting protein 1 | clathrin-coated vesicle, clathrin-coated vesicle membrane, cytoplasm, cytoskeleton, cytosol, extrinsic component of cytoplasmic side of plasma membrane, extrinsic component of postsynaptic membrane, extrinsic component of presynaptic membrane, glutamatergic synapse, Golgi apparatus, intracellular membrane-bounded organelle, membrane, nucleus, postsynapse, presynapse, | actin filament binding, AP-2 adaptor complex binding, clathrin adaptor activity, clathrin binding, clathrin light chain binding, epidermal growth factor receptor binding, glutamate receptor binding, phosphatidylinositol binding, phosphatidylinositol-3,4-bisphosphate binding, phosphatidylinositol-3,5-bisphosphate binding, phosphatidylinositol-3-phosphate binding, protein heterodimerization activity, protein homodimerization activity, structural constituent of cytoskeleton, | regulation of endocytosis |
| 1957 | 185:1512176-1513176 | Insyn2a | Inhibitory synaptic factor 2A | postsynaptic density, |  | inhibitory postsynaptic potential |
| 2060 | 313:443210-444210 | FRY | Protein furry homolog | cell cortex, microtubule organizing center, site of polarized growth, spindle pole, | enzyme inhibitor activity, | neuron projection development |
| 2261 | 302:576353-577353 | med12 | Mediator of RNA polymerase II transcription subunit 12 | mediator complex, | beta-catenin binding, DNA-binding transcription factor activity, transcription coactivator activity, | ventricular system development |
| 2345 | 305:616438-617438 | NECTIN1 | Nectin-1 | adherens junction, apical junction complex, cell-cell contact zone, dendrite, extracellular region, growth cone membrane, hippocampal mossy fiber to CA3 synapse, integral component of membrane, integral component of presynaptic active zone membrane, intracellular membrane-bounded organelle, membrane, plasma membrane, | carbohydrate binding, cell adhesion molecule binding, coreceptor activity, identical protein binding, protein homodimerization activity, protein-containing complex binding, virion binding, virus receptor activity, | virion attachment to host cell |
| 2437 | 371:565888-566888 | cdh2 | Cadherin-2 | apical part of cell, catenin complex, cytoplasm, integral component of plasma membrane, integral component of postsynaptic specialization membrane, integral component of presynaptic active zone membrane, intercalated disc, lamellipodium, neuron projection, perinuclear region of cytoplasm, plasma membrane, postsynaptic density, presynaptic membrane, | cadherin binding, calcium ion binding, | ventricular system development |
| 2481 | 363:566722-567722 | mical3a | Protein-methionine sulfoxide oxidase mical3a | cytoplasm, cytoskeleton, nucleus, | actin binding, actin filament binding, FAD binding, metal ion binding, oxidoreductase activity, acting on paired donors, with incorporation or reduction of molecular oxygen, NAD(P)H as one donor, and incorporation of one atom of oxygen, small GTPase binding, | exocytosis |
| 2576 | 415:46979-47979 | phb2 | Prohibitin-2 | cell surface, cytoplasm, mitochondrial inner membrane, mitochondrial prohibitin complex, mitochondrion, nuclear matrix, nucleus, plasma membrane, | protein homodimerization activity, | mitophagy |
| 2669 | 445:73934-74934 | ZXDC | Zinc finger protein ZXDC | nucleus, | C2H2 zinc finger domain binding, LRR domain binding, metal ion binding, transcription coactivator activity, transcription coregulator activity, | regulation of transcription by RNA polymerase II |
| 2758 | 450:33363-34363 | Pgm3 | Phosphoacetylglucosamine mutase |  | magnesium ion binding, phosphoacetylglucosamine mutase activity, phosphoglucomutase activity, | UDP-N-acetylglucosamine biosynthetic process |
| 2912 | 443:468283-469283 | - | - |  |  | BP: |
| 3198 | 536:115434-116434 | SLC43A3 | Equilibrative nucleobase transporter 1 | basolateral plasma membrane, integral component of membrane, | adenine transmembrane transporter activity, fatty acid transmembrane transporter activity, guanine transmembrane transporter activity, xenobiotic transmembrane transporter activity, | hypoxanthine transport |
| 3358 | 510:74439-75439 | Wdr19 | WD repeat-containing protein 19 | cilium, cytoplasm, cytoskeleton, intraciliary transport particle A, motile cilium, non-motile cilium, photoreceptor connecting cilium, photoreceptor outer segment, |  | smoothened signaling pathway involved in dorsal/ventral neural tube patterning |
| 3463 | 570:58908-59908 | KLHDC4 | Kelch domain-containing protein 4 |  |  | BP: |
| 3554 | 578:564979-565979 | Fbxl3 | F-box/LRR-repeat protein 3 | cytosol, nuclear body, nucleoplasm, nucleus, SCF ubiquitin ligase complex, | ubiquitin-protein transferase activity, | SCF-dependent proteasomal ubiquitin-dependent protein catabolic process |
| 3589 | 607:339200-340200 | lmbr1 | Limb region 1 protein homolog | integral component of plasma membrane, | transmembrane signaling receptor activity, | signal transduction |
| 3642 | 637:319293-320293 | farsa | Phenylalanine--tRNA ligase alpha subunit | cytoplasm, phenylalanine-tRNA ligase complex, | ATP binding, phenylalanine-tRNA ligase activity, tRNA binding, | protein heterotetramerization |
| 3766 | 671:41424-42424 | CC2D1A | Coiled-coil and C2 domain-containing protein 1A | cytosol, extracellular exosome, fibrillar center, membrane, microtubule organizing center, nucleus, plasma membrane, | cadherin binding, DNA-binding transcription factor activity, RNA polymerase II-specific, DNA-binding transcription repressor activity, RNA polymerase II-specific, RNA polymerase II cis-regulatory region sequence-specific DNA binding, | regulation of transcription by RNA polymerase II |
| 3911 | 709:55068-56068 | MPRIP | Myosin phosphatase Rho-interacting protein | actin cytoskeleton, cytosol, focal adhesion, | actin filament binding, cadherin binding, | actin filament organization |
| 4119 | 744:374346-375346 | ATP6V1A | V-type proton ATPase catalytic subunit A | cytosol, plasma membrane, transport vesicle, vacuolar proton-transporting V-type ATPase, V1 domain, | ATP binding, proton-transporting ATP synthase activity, rotational mechanism, proton-transporting ATPase activity, rotational mechanism, | proton transmembrane transport |
| 4123 | 691:14283-15283 | Abca3 | Phospholipid-transporting ATPase ABCA3 | alveolar lamellar body, alveolar lamellar body membrane, cytoplasmic vesicle membrane, integral component of membrane, intracellular membrane-bounded organelle, lamellar body, lamellar body membrane, late endosome, lysosomal membrane, multivesicular body membrane, plasma membrane, | ABC-type xenobiotic transporter activity, ATP binding, ATP hydrolysis activity, ATPase-coupled transmembrane transporter activity, lipid transporter activity, phosphatidylcholine flippase activity, phosphatidylcholine transfer activity, | xenobiotic transport |
| 4136 | 707:24508-25508 | KDM5A | Lysine-specific demethylase 5A | nucleolus, nucleoplasm, nucleus, protein-DNA complex, | chromatin DNA binding, DNA binding, histone binding, histone demethylase activity, histone H3-tri/di/monomethyl-lysine-4 demethylase activity, methylated histone binding, transcription cis-regulatory region binding, transcription coactivator activity, zinc ion binding, | regulation of DNA-binding transcription factor activity |
| 4263 | 762:172258-173258 | Sbf1 | Myotubularin-related protein 5 | cytoplasm, nuclear body, perinuclear region of cytoplasm, | guanyl-nucleotide exchange factor activity, pseudophosphatase activity, | spermatogenesis |
| 4270 | 762:369807-370807 | NET1 | Neuroepithelial cell-transforming gene 1 protein | cytosol, nucleus, | guanyl-nucleotide exchange factor activity, | signal transduction |
| 4506 | 748:1106286-1107286 | GGNBP2 | Gametogenetin-binding protein 2 | cytoplasm, nucleus, |  | spermatogenesis |
| 4652 | 797:150139-151139 | Nmt1 | Glycylpeptide N-tetradecanoyltransferase 1 | cytoplasm, cytosol, extrinsic component of membrane, plasma membrane, | glycylpeptide N-tetradecanoyltransferase activity, myristoyltransferase activity, | N-terminal protein myristoylation |
| 4954 | 886:22299-23299 | FRAS1 | Extracellular matrix organizing protein FRAS1 | basement membrane, integral component of membrane, plasma membrane, | extracellular matrix structural constituent, metal ion binding, | skin development |
| 5456 | 989:821297-822297 | Capn15 | Calpain-15 | cytoplasm, | calcium-dependent cysteine-type endopeptidase activity, metal ion binding, | proteolysis |
| 5688 | 997:435787-436787 | PLEKHH2 | Pleckstrin homology domain-containing family H member 2 | cortical actin cytoskeleton, cytoplasm, cytosol, lamellipodium, nuclear body, nucleoplasm, plasma membrane, | actin binding, | negative regulation of actin filament depolymerization |
| 5735 | 609:77255-78255 | ADCYAP1R1 | Pituitary adenylate cyclase-activating polypeptide type I receptor | bicellular tight junction, caveola, cell surface, endosome, integral component of plasma membrane, intracellular membrane-bounded organelle, neuron projection, plasma membrane, receptor complex, rough endoplasmic reticulum, | adenylate cyclase binding, G protein-coupled peptide receptor activity, neuropeptide binding, peptide hormone binding, signaling receptor activity, small GTPase binding, vasoactive intestinal polypeptide receptor activity, | spermatogenesis |
| 5814 | 1080:538622-539622 | scamp5 | Secretory carrier-associated membrane protein 5 | Golgi membrane, integral component of membrane, plasma membrane, recycling endosome membrane, synaptic vesicle membrane, trans-Golgi network membrane, |  | protein transport |
| 5953 | 1064:295106-296106 | zpld1 | Zona pellucida-like domain-containing protein 1 | collagen-containing extracellular matrix, cytoplasmic vesicle membrane, extracellular region, integral component of membrane, |  |  |
| 6028 | 1104:266479-267479 | CACNA1D | Voltage-dependent L-type calcium channel subunit alpha-1D | voltage-gated calcium channel complex, | metal ion binding, voltage-gated calcium channel activity, | regulation of ion transmembrane transport |
| 6157 | 1105:500302-501302 | SLC7A8 | Large neutral amino acids transporter small subunit 2 | apical plasma membrane, basolateral plasma membrane, cytoplasm, integral component of membrane, microvillus membrane, | amino acid transmembrane transporter activity, glycine transmembrane transporter activity, L-alanine transmembrane transporter activity, L-leucine transmembrane transporter activity, organic cation transmembrane transporter activity, peptide antigen binding, thyroid hormone transmembrane transporter activity, toxin transmembrane transporter activity, | valine transport |
| 6183 | 1144:197128-198128 | notch1 | Neurogenic locus notch homolog protein 1 | integral component of membrane, nucleus, plasma membrane, | calcium ion binding, Notch binding, signaling receptor activity, | regulation of transcription, DNA-templated |
| 6224 | 1150:16354-17354 | nup133 | Nuclear pore complex protein Nup133 | kinetochore, nuclear pore outer ring, | structural constituent of nuclear pore, | transcription-dependent tethering of RNA polymerase II gene DNA at nuclear periphery |
| 6340 | 1189:232346-233346 | Mapt | Microtubule-associated protein tau | axolemma, axon, axonal growth cone, axoneme, cell body, cytoplasm, cytoplasmic ribonucleoprotein granule, cytoplasmic side of plasma membrane, cytosol, dendrite, extracellular region, glial cell projection, growth cone, main axon, membrane raft, microtubule, microtubule cytoskeleton, neurofibrillary tangle, neuron projection, neuronal cell body, nuclear periphery, nuclear speck, nucleus, plasma membrane, postsynaptic density, somatodendritic compartment, tubulin complex, | apolipoprotein binding, chaperone binding, DNA binding, enzyme binding, heat shock protein binding, Hsp90 protein binding, identical protein binding, lipoprotein particle binding, microtubule binding, microtubule lateral binding, protein kinase binding, protein phosphatase 2A binding, protein-containing complex binding, SH3 domain binding, | synapse organization |
| 6497 | 1106:26063-27063 | COX7C | Cytochrome c oxidase subunit 7C, mitochondrial | integral component of membrane, mitochondrial respiratory chain complex IV, respiratory chain complex IV, |  | mitochondrial electron transport, cytochrome c to oxygen |
| 6732 | 1278:153993-154993 | PTPRG | Receptor-type tyrosine-protein phosphatase gamma | extracellular exosome, integral component of plasma membrane, | identical protein binding, protein tyrosine phosphatase activity, transmembrane receptor protein tyrosine phosphatase activity, | transmembrane receptor protein tyrosine kinase signaling pathway |
| 6869 | 1300:204077-205077 | CGNL1 | Cingulin-like protein 1 | bicellular tight junction, myosin complex, protein-containing complex, |  | protein localization to cell-cell junction |
| 6972 | 1304:138738-139738 | CYFIP1 | Cytoplasmic FMR1-interacting protein 1 | axonal growth cone, central region of growth cone, cytosol, dendritic growth cone, dendritic spine, excitatory synapse, extracellular exosome, extracellular region, filopodium tip, focal adhesion, lamellipodium, mRNA cap binding complex, neuron projection, neuronal cell body, perinuclear region of cytoplasm, peripheral region of growth cone, ruffle, SCAR complex, secretory granule lumen, specific granule lumen, synapse, terminal bouton, tertiary granule lumen, | actin filament binding, RNA 7-methylguanosine cap binding, small GTPase binding, translation regulator activity, | ruffle organization |
| 7092 | 1355:160678-161678 | psmd11a | 26S proteasome non-ATPase regulatory subunit 11A | cytosol, nucleus, proteasome accessory complex, proteasome regulatory particle, lid subcomplex, | structural molecule activity, | ubiquitin-dependent protein catabolic process |
| 7201 | 1329:595788-596788 | Prpf6 | Pre-mRNA-processing factor 6 | catalytic step 2 spliceosome, nuclear speck, nucleus, U2-type precatalytic spliceosome, U4/U6 x U5 tri-snRNP complex, U5 snRNP, | androgen receptor binding, identical protein binding, ribonucleoprotein complex binding, RNA binding, transcription coactivator activity, | spliceosomal tri-snRNP complex assembly |
| 7286 | 1417:33562-34562 | MTTP | Microsomal triglyceride transfer protein | endoplasmic reticulum, | ceramide 1-phosphate transfer activity, lipid binding, protein heterodimerization activity, | triglyceride transport |
| 7488 | 1457:138799-139799 | Slc12a7 | Solute carrier family 12 member 7 | integral component of plasma membrane, protein-containing complex, synapse, | ammonium transmembrane transporter activity, potassium:chloride symporter activity, protein kinase binding, | potassium ion import across plasma membrane |
| 7514 | 1475:48921-49921 | VPS54 | Vacuolar protein sorting-associated protein 54 | cytosol, GARP complex, Golgi apparatus, membrane, nucleoplasm, perinuclear region of cytoplasm, trans-Golgi network, trans-Golgi network membrane, | syntaxin binding, | retrograde transport, endosome to Golgi |
| 7581 | 1471:68482-69482 | ATP6V1E1 | V-type proton ATPase subunit E 1 | apical plasma membrane, cytosol, endosome, vacuolar proton-transporting V-type ATPase, V1 domain, | hydrolase activity, proton-transporting ATPase activity, rotational mechanism, | proton transmembrane transport |
| 7601 | 1505:62364-63364 | MTSS1 | Protein MTSS 1 | actin cytoskeleton, cytoplasm, endocytic vesicle, intrinsic component of the cytoplasmic side of the plasma membrane, ruffle, | actin binding, actin monomer binding, identical protein binding, phospholipid binding, signaling receptor binding, | transmembrane receptor protein tyrosine kinase signaling pathway |
| 7654 | 1472:165808-166808 | Galnt18 | Polypeptide N-acetylgalactosaminyltransferase 18 | Golgi apparatus, Golgi membrane, integral component of membrane, | carbohydrate binding, metal ion binding, polypeptide N-acetylgalactosaminyltransferase activity, | protein O-linked glycosylation |
| 7676 | 1459:380385-381385 | Ube2o | (E3-independent) E2 ubiquitin-conjugating enzyme UBE2O | cytoplasm, cytosol, nuclear body, nucleoplasm, nucleus, | ATP binding, cysteine-type endopeptidase inhibitor activity, ubiquitin conjugating enzyme activity, ubiquitin protein ligase activity, ubiquitin-protein transferase activity, | retrograde transport, endosome to Golgi |
| 7683 | 1538:246444-247444 | Nfix | Nuclear factor 1 X-type | nucleus, | DNA-binding transcription activator activity, RNA polymerase II-specific, DNA-binding transcription factor activity, RNA polymerase II-specific, RNA polymerase II cis-regulatory region sequence-specific DNA binding, RNA polymerase II transcription regulatory region sequence-specific DNA binding, sequence-specific double-stranded DNA binding, | regulation of transcription by RNA polymerase II |
| 7707 | 1537:145993-146993 | Rims2 | Regulating synaptic membrane exocytosis protein 2 | cytoskeleton of presynaptic active zone, GABA-ergic synapse, glutamatergic synapse, inhibitory synapse, neuron projection, photoreceptor ribbon synapse, presynaptic active zone cytoplasmic component, presynaptic membrane, protein-containing complex, synapse, | metal ion binding, protein domain specific binding, protein-containing complex binding, small GTPase binding, structural constituent of presynaptic active zone, transmembrane transporter binding, | synaptic vesicle priming |
| 7714 | 1537:230717-231717 | ints3 | Integrator complex subunit 3 | cytoplasm, nucleus, SOSS complex, |  | response to ionizing radiation |
| 7881 | 1586:43438-44438 | rfx4 | Transcription factor RFX4 | nucleus, | DNA-binding transcription factor activity, RNA polymerase II-specific, RNA polymerase II cis-regulatory region sequence-specific DNA binding, | ventral midline development |
| 8027 | 1590:144284-145284 | STT3B | Dolichyl-diphosphooligosaccharide--protein glycosyltransferase subunit STT3B | integral component of membrane, oligosaccharyltransferase I complex, oligosaccharyltransferase II complex, | dolichyl-diphosphooligosaccharide-protein glycotransferase activity, metal ion binding, | ubiquitin-dependent ERAD pathway |
| 8154 | 1681:36938-37938 | ZFPM1 | Zinc finger protein ZFPM1 | chromatin, nucleoplasm, nucleus, transcription regulator complex, transcription repressor complex, | DNA binding, metal ion binding, RNA polymerase II-specific DNA-binding transcription factor binding, transcription corepressor activity, | ventricular septum morphogenesis |
| 8204 | 1604:503879-504879 | loxl3b | Lysyl oxidase homolog 3B | cytoplasm, extracellular space, membrane, nucleus, | copper ion binding, fibronectin binding, protein-lysine 6-oxidase activity, scavenger receptor activity, | spinal cord development |
| 8223 | 1608:524884-525884 | DSCAM | Down syndrome cell adhesion molecule | axon, dendrite, extracellular region, growth cone, integral component of plasma membrane, membrane, neuronal cell body, plasma membrane, synapse, | cell-cell adhesion mediator activity, netrin receptor binding, protein tyrosine kinase binding, | synapse assembly |
| 8407 | 1638:422085-423085 | RPS4 | 40S ribosomal protein S4 | cytosolic small ribosomal subunit, | RNA binding, rRNA binding, structural constituent of ribosome, | translation |
| 8431 | 1725:199737-200737 | OPLAH | 5-oxoprolinase |  | 5-oxoprolinase (ATP-hydrolyzing) activity, ATP binding, | glutathione metabolic process |
| 8491 | 1771:183440-184440 | MON1B | Vacuolar fusion protein MON1 homolog B | cytoplasm, Mon1-Ccz1 complex, |  | vesicle-mediated transport |
| 8498 | 1771:496770-497770 | ABHD14B | Putative protein-lysine deacylase ABHD14B | cytoplasm, cytosol, extracellular exosome, nucleolus, nucleoplasm, nucleus, | hydrolase activity, | positive regulation of transcription by RNA polymerase II |
| 8627 | 1616:21483-22483 | mak16 | Protein MAK16 homolog | nucleolus, preribosome, large subunit precursor, |  | maturation of LSU-rRNA |
| 8959 | 1990:156204-157204 | Rprd1b | Regulation of nuclear pre-mRNA domain-containing protein 1B | nucleoplasm, nucleus, RNA polymerase II, holoenzyme, | identical protein binding, RNA polymerase II complex binding, | regulation of cell cycle process |
| 8974 | 1959:291143-292143 | Huwe1 | E3 ubiquitin-protein ligase HUWE1 | cytoplasm, cytosol, Golgi membrane, mitochondrion, nucleoplasm, nucleus, | DNA binding, ubiquitin protein ligase activity, ubiquitin-protein transferase activity, | protein ubiquitination |
| 9172 | 2010:169408-170408 | ISY1 | Pre-mRNA-splicing factor ISY1 homolog | catalytic step 2 spliceosome, nucleoplasm, nucleus, post-mRNA release spliceosomal complex, post-spliceosomal complex, Prp19 complex, U2-type catalytic step 1 spliceosome, | RNA binding, | mRNA splicing, via spliceosome |
| 9501 | 2180:110935-111935 | Parp4 | Protein mono-ADP-ribosyltransferase PARP4 | cytoplasm, cytosol, nucleoplasm, nucleus, spindle, spindle microtubule, | enzyme binding, NAD+ ADP-ribosyltransferase activity, protein ADP-ribosylase activity, | regulation of telomerase activity |
| 9594 | 2195:7382-8382 | Kcnh7 | Potassium voltage-gated channel subfamily H member 7 | integral component of plasma membrane, | inward rectifier potassium channel activity, potassium channel activity, protein-containing complex binding, voltage-gated potassium channel activity, | regulation of membrane potential |
| 9900 | 2412:16858-17858 | fgfr4 | Fibroblast growth factor receptor 4 | endoplasmic reticulum, endosome, integral component of plasma membrane, receptor complex, | ATP binding, fibroblast growth factor binding, fibroblast growth factor-activated receptor activity, protein serine/threonine/tyrosine kinase activity, transmembrane receptor protein tyrosine kinase activity, | transmembrane receptor protein tyrosine kinase signaling pathway |
| 10075 | 2494:173683-174683 | WNK2 | Serine/threonine-protein kinase WNK2 | cytoplasm, cytosol, plasma membrane, | ATP binding, chloride channel inhibitor activity, potassium channel inhibitor activity, protein serine kinase activity, protein serine/threonine kinase activity, protein serine/threonine/tyrosine kinase activity, | protein phosphorylation |
| 10363 | 2616:382350-383350 | Atp10a | Phospholipid-transporting ATPase VA | endoplasmic reticulum, endoplasmic reticulum membrane, integral component of plasma membrane, phospholipid-translocating ATPase complex, plasma membrane, | ATP binding, ATP hydrolysis activity, ATPase-coupled intramembrane lipid transporter activity, glycosylceramide flippase activity, magnesium ion binding, phosphatidylcholine flippase activity, phosphatidylcholine floppase activity, | positive regulation of membrane tubulation |
| 10734 | 2899:121910-122910 | SPTAN1 | Spectrin alpha chain, non-erythrocytic 1 | cell junction, cell projection, cortical actin cytoskeleton, costamere, plasma membrane, | actin filament binding, calcium ion binding, calmodulin binding, | actin filament capping |
| 10960 | 3002:50929-51929 | TECPR1 | Tectonin beta-propeller repeat-containing protein 1 | autophagosome membrane, cytoplasmic vesicle, integral component of membrane, lysosomal membrane, | phosphatidylinositol-3-phosphate binding, | autophagy |
| 11102 | 3114:160782-161782 | sh3bp4 | SH3 domain-binding protein 4 | clathrin-coated pit, clathrin-coated vesicle, cytoplasm, nucleus, | GDP-dissociation inhibitor activity, small GTPase binding, | regulation of catalytic activity |
| 11221 | 3182:23266-24266 | Mark2 | Serine/threonine-protein kinase MARK2 | actin filament, basal cortex, cytoplasm, dendrite, lateral plasma membrane, membrane, microtubule bundle, nucleus, plasma membrane, | ATP binding, lipid binding, magnesium ion binding, protein N-terminus binding, protein serine kinase activity, protein serine/threonine kinase activity, protein serine/threonine/tyrosine kinase activity, tau protein binding, tau-protein kinase activity, | Wnt signaling pathway |
| 11439 | 3425:75430-76430 | TEKT3 | Tektin-3 | acrosomal membrane, extracellular exosome, microtubule cytoskeleton, nucleus, outer acrosomal membrane, sperm flagellum, |  | regulation of brood size |
| 11461 | 3498:33329-34230 | Pias2 | E3 SUMO-protein ligase PIAS2 | nuclear speck, nucleus, PML body, | androgen receptor binding, DNA binding, estrogen receptor binding, glucocorticoid receptor binding, protein domain specific binding, RNA polymerase II-specific DNA-binding transcription factor binding, SUMO ligase activity, SUMO transferase activity, transcription coregulator activity, ubiquitin protein ligase binding, zinc ion binding, | transcription, DNA-templated |
| 11524 | 3532:82805-83805 | CAPN1 | Calpain-1 catalytic subunit | cornified envelope, cytoplasm, cytosol, extracellular exosome, extracellular region, ficolin-1-rich granule lumen, focal adhesion, lysosome, membrane, mitochondrion, plasma membrane, | calcium ion binding, calcium-dependent cysteine-type endopeptidase activity, peptidase activity, | self proteolysis |
| 11750 | 3629:138048-139048 | lmna | Lamin-A | intermediate filament, nuclear envelope, |  |  |
| 11765 | 3758:15954-16954 | dye | Nuclear pore complex protein Nup93 | nuclear membrane, nuclear periphery, nuclear pore, | structural constituent of nuclear pore, | protein import into nucleus |
| 12010 | 4053:92573-93573 | B3gntl1 | UDP-GlcNAc:betaGal beta-1,3-N-acetylglucosaminyltransferase-like protein 1 |  | glycosyltransferase activity, |  |
| 12199 | 4205:80848-81848 | Nr2f2 | COUP transcription factor 2 | cytosol, nucleoplasm, nucleus, | DNA binding, DNA-binding transcription factor activity, nuclear receptor activity, protein homodimerization activity, retinoic acid binding, RNA polymerase II cis-regulatory region sequence-specific DNA binding, sequence-specific DNA binding, zinc ion binding, | trophoblast giant cell differentiation |
| 12204 | 4211:9634-10634 | ARFGEF3 | Brefeldin A-inhibited guanine nucleotide-exchange protein 3 | integral component of membrane, transport vesicle membrane, | guanyl-nucleotide exchange factor activity, | regulation of ARF protein signal transduction |
| 12236 | 4264:23949-24949 | DIPK1A | Divergent protein kinase domain 1A | endoplasmic reticulum membrane, integral component of membrane, |  |  |
| 12246 | 4282:30989-31965 | POFUT2 | GDP-fucose protein O-fucosyltransferase 2 | endoplasmic reticulum, Golgi apparatus, | peptide-O-fucosyltransferase activity, | protein O-linked fucosylation |
| 12262 | 3928:34505-35505 | RAB33B | Ras-related protein Rab-33B | Golgi lumen, Golgi membrane, | GTP binding, GTPase activity, | skeletal system morphogenesis |
| 12393 | 4423:96309-97309 | Necap1 | Adaptin ear-binding coat-associated protein 1 | clathrin vesicle coat, clathrin-coated pit, |  | vesicle-mediated transport |
| 12561 | 4599:22491-23491 | TAOK3 | Serine/threonine-protein kinase TAO3 | cytoplasm, | ATP binding, protein serine kinase activity, protein serine/threonine kinase activity, protein serine/threonine/tyrosine kinase activity, transferase activity, | regulation of MAPK cascade |
| 12562 | 4599:39360-40360 | Cdipt | CDP-diacylglycerol--inositol 3-phosphatidyltransferase | endoplasmic reticulum membrane, Golgi apparatus, integral component of membrane, plasma membrane, | alcohol binding, carbohydrate binding, CDP-diacylglycerol-inositol 3-phosphatidyltransferase activity, diacylglycerol binding, manganese ion binding, | phosphatidylinositol biosynthetic process |
| 12905 | 5574:5017-6017 | FLNC | Filamin-C | costamere, cytoplasm, cytoskeleton, cytosol, focal adhesion, plasma membrane, sarcolemma, Z disc, | actin filament binding, ankyrin binding, cytoskeletal protein binding, identical protein binding, | sarcomere organization |
| 13065 | 5832:40096-41096 | HSPD1 | 60 kDa heat shock protein, mitochondrial | cytoplasm, mitochondrial matrix, plasma membrane, protein-containing complex, secretory granule, | ATP binding, ATP hydrolysis activity, isomerase activity, lipopolysaccharide binding, | T cell activation |
| 13069 | 5832:82302-83302 | sf3b1 | Splicing factor 3B subunit 1 | nucleus, spliceosomal complex, | mRNA binding, | spliceosomal complex assembly |
| 13285 | 6505:23227-24227 | Tbc1d9b | TBC1 domain family member 9B | integral component of membrane, | calcium ion binding, GTPase activator activity, | activation of GTPase activity |
| 13300 | 6520:17284-18284 | DNAH11 | Dynein axonemal heavy chain 11 | 9+2 motile cilium, axoneme, dynein complex, microtubule, motile cilium, | ATP binding, dynein intermediate chain binding, dynein light intermediate chain binding, minus-end-directed microtubule motor activity, | microtubule-based movement |
| 13375 | 6821:57002-58002 | TNNT1 | Troponin T, slow skeletal muscle | cytosol, troponin complex, | tropomyosin binding, troponin T binding, | transition between fast and slow fiber |
| 13475 | 6937:104345-105345 | RFC2 | Replication factor C subunit 2 | Ctf18 RFC-like complex, DNA replication factor C complex, nucleoplasm, nucleus, | ATP binding, ATP hydrolysis activity, DNA binding, enzyme binding, | positive regulation of DNA-directed DNA polymerase activity |
| 13575 | 7552:46654-47654 | Rarg | Retinoic acid receptor gamma | chromatin, cytoplasm, nucleoplasm, nucleus, transcription regulator complex, | chromatin binding, DNA binding, DNA-binding transcription factor activity, nuclear receptor activity, retinoid X receptor binding, RNA polymerase II cis-regulatory region sequence-specific DNA binding, sequence-specific double-stranded DNA binding, zinc ion binding, | trachea cartilage development |
| 13587 | 7502:6473-7473 | fgfr1a | Fibroblast growth factor receptor 1-A | cytoplasmic vesicle, cytosol, integral component of plasma membrane, nucleus, plasma membrane, receptor complex, | ATP binding, fibroblast growth factor binding, fibroblast growth factor-activated receptor activity, heparin binding, protein serine/threonine/tyrosine kinase activity, transmembrane receptor protein tyrosine kinase activity, | transmembrane receptor protein tyrosine kinase signaling pathway |
| 13626 | 7835:3245-4245 | Plekhg2 | Pleckstrin homology domain-containing family G member 2 |  | guanyl-nucleotide exchange factor activity, | regulation of actin filament polymerization |
| 13760 | 8833:11992-12992 | VSTM2L | V-set and transmembrane domain-containing protein 2-like protein | axon, cytoplasm, extracellular region, plasma membrane, | cell-cell adhesion mediator activity, | negative regulation of neuron apoptotic process |
| 13988 | 11086:9106-10106 | DDX18 | ATP-dependent RNA helicase DDX18 | chromosome, membrane, nucleolus, | ATP binding, ATP hydrolysis activity, RNA binding, RNA helicase activity, | maturation of LSU-rRNA from tricistronic rRNA transcript (SSU-rRNA, 5.8S rRNA, LSU-rRNA) |
| 14005 | 11382:13312-14312 | Kif5b | Kinesin-1 heavy chain | axon cytoplasm, axonal growth cone, ciliary rootlet, cytoplasm, dendrite cytoplasm, endocytic vesicle, kinesin complex, lysosomal membrane, membrane-bounded organelle, microtubule, microtubule cytoskeleton, neuron projection, perinuclear region of cytoplasm, phagocytic vesicle, vesicle, | ATP binding, ATP hydrolysis activity, identical protein binding, JUN kinase binding, microtubule binding, microtubule lateral binding, microtubule motor activity, plus-end-directed microtubule motor activity, | vesicle transport along microtubule |
| 14019 | 11473:8706-9706 | THBS2 | Thrombospondin-2 | collagen-containing extracellular matrix, extracellular region, | calcium ion binding, heparin binding, | negative regulation of angiogenesis |
| 14252 | 14643:4198-5198 | GLUD2 | Glutamate dehydrogenase 2, mitochondrial | mitochondrial matrix, mitochondrion, | ADP binding, glutamate dehydrogenase (NAD+) activity, glutamate dehydrogenase (NADP+) activity, glutamate dehydrogenase [NAD(P)+] activity, GTP binding, leucine binding, | glutamate metabolic process |
| 14285 | 18354:29293-30293 | arnt2 | Aryl hydrocarbon receptor nuclear translocator 2 | aryl hydrocarbon receptor complex, cytoplasm, nucleus, transcription regulator complex, | DNA-binding transcription factor activity, DNA-binding transcription factor activity, RNA polymerase II-specific, protein heterodimerization activity, RNA polymerase II cis-regulatory region sequence-specific DNA binding, | ventricular system development |
| 14305 | 15417:8409-9409 | Pde9a | High affinity cGMP-specific 3',5'-cyclic phosphodiesterase 9A | cytosol, endoplasmic reticulum, Golgi apparatus, nucleoplasm, perikaryon, perinuclear region of cytoplasm, plasma membrane, ruffle membrane, sarcolemma, | 3\\,5\\-cyclic-GMP phosphodiesterase activity, 3\\,5\\-cyclic-nucleotide phosphodiesterase activity, identical protein binding, metal ion binding, | signal transduction |
| 14444 | C21501449:1-161 | Sorcs2 | VPS10 domain-containing receptor SorCS2 | cytosol, dendritic spine, early endosome membrane, integral component of membrane, integral component of plasma membrane, perikaryon, postsynaptic density, postsynaptic density membrane, recycling endosome membrane, |  | long-term synaptic depression |

References for Supplemental Material

Attard, C. R., C. J. Brauer, J. Sandoval‐Castillo, L. K. Faulks, P. J. Unmack, D. M. Gilligan, and L. B. Beheregaray. 2018. Ecological disturbance influences adaptive divergence despite high gene flow in golden perch (Macquaria ambigua): implications for management and resilience to climate change. Molecular Ecology **27**:196-215.

Brauer, C. J., M. P. Hammer, and L. B. Beheregaray. 2016. Riverscape genomics of a threatened fish across a hydroclimatically heterogeneous river basin. Molecular Ecology **25**:5093-5113.

Brauer, C. J., P. J. Unmack, S. Smith, L. Bernatchez, and L. B. Beheregaray. 2018. On the roles of landscape heterogeneity and environmental variation in determining population genomic structure in a dendritic system. Molecular Ecology **27**:3484-3497.

Claude, J. 2008. Morphometrics with R. Springer Science & Business Media, New York, NY, USA.

Coop, G., D. Witonsky, A. Di Rienzo, and J. K. Pritchard. 2010. Using environmental correlations to identify loci underlying local adaptation. Genetics **185**:1411-1423.

Crossman, S., and O. Li. 2015. Surface Hydrology Lines (National). Page <http://pid.geoscience.gov.au/dataset/ga/83130> Geoscience Australia, Canberra. Accessed June, 2017.

Danecek, P., A. Auton, G. Abecasis, C. A. Albers, E. Banks, M. A. DePristo, R. E. Handsaker, G. Lunter, G. T. Marth, S. T. Sherry, G. McVean, R. Durbin, and G. Genomes Project Analysis. 2011. The variant call format and VCFtools. Bioinformatics (Oxford, England) **27**:2156-2158.

ESRI. 2011. ArcGIS Desktop: Release 10. Redlands, CA: Environmental Systems Research Institute.

Forester, B. R., J. R. Lasky, H. H. Wagner, and D. L. Urban. 2018. Comparing methods for detecting multilocus adaptation with multivariate genotype–environment associations. Molecular Ecology **27**:2215-2233.

Gautier, M. 2015. Genome-wide scan for adaptive divergence and association with population-specific covariates. Genetics **201**:1555-1579.

Gu, Z., L. Gu, R. Eils, M. Schlesner, and B. Brors. 2014. Circlize implements and enhances circular visualization in R. Bioinformatics **30**:2811-2812.

Günther, T., and G. Coop. 2013. Robust identification of local adaptation from allele frequencies. Genetics **195**:205-220.

Ho, W.-C., Y. Ohya, and J. Zhang. 2017. Testing the neutral hypothesis of phenotypic evolution. Proceedings of the National Academy of Sciences **114**:12219-12224.

Höllinger, I., P. S. Pennings, and J. Hermisson. 2019. Polygenic adaptation: From sweeps to subtle frequency shifts. PLoS genetics **15**:e1008035.

Jombart, T. 2008. adegenet: a R package for the multivariate analysis of genetic markers. Bioinformatics **24**:1403-1405.

Lischer, H. E., and L. Excoffier. 2012. PGDSpider: an automated data conversion tool for connecting population genetics and genomics programs. Bioinformatics **28**:298-299.

McGuigan, K., S. Chenoweth, xa, F, M. Blows, xa, W, and M. C. Associate Editor: James. 2005. Phenotypic Divergence along Lines of Genetic Variance. The American Naturalist **165**:32-43.

McGuigan, K., C. E. Franklin, C. Moritz, and M. W. Blows. 2003. Adaptation of rainbow fish to lake and stream habitats. Evolution **57**:104-118.

Mitchell-Olds, T., J. H. Willis, and D. B. Goldstein. 2007. Which evolutionary processes influence natural genetic variation for phenotypic traits? Nature Reviews Genetics **8**:845-856.

Mussmann, S. M., M. R. Douglas, T. K. Chafin, and M. E. Douglas. 2019. BA3‐SNPs: Contemporary migration reconfigured in BayesAss for next‐generation sequence data. Methods in Ecology and Evolution **10**:1808-1813.

Oksanen, J., F. G. Blanchet, M. Friendly, R. Kindt, P. Legendre, D. McGlinn, P. R. Minchin, R. O’Hara, G. L. Simpson, P. Solymos, M. H. H. Stevens, E. Szoecs, and H. Wagner. 2019. vegan: Community Ecology Package. R package version 2.5-6.

Paradis, E., and K. Schliep. 2019. ape 5.0: an environment for modern phylogenetics and evolutionary analyses in R. Bioinformatics **35**:526-528.

Pina-Martins, F. 2016. geste2baypass. <https://github.com/CoBiG2/RAD_Tools/blob/master/geste2baypass.py>

Raj, A., M. Stephens, and J. K. Pritchard. 2014. fastSTRUCTURE: variational inference of population structure in large SNP data sets. Genetics **197**:573-589.

RC Team. 2019. R: A language and environment for statistical computing. Pages <https://www.R-project.org/>. R Foundation for Statistical Computing, Vienna, Austria.

Rosenberg, N. A. 2004. DISTRUCT: a program for the graphical display of population structure. Molecular Ecology Notes **4**:137-138.

Stein, J. L., Hutchison, M.F., Stein, J.A. . 2011. National Environmental Stream Attributes v1.1.3. Page <http://pid.geoscience.gov.au/dataset/ga/73045> Geoscience Australia, Canberra. Accessed June, 2017.

Sunnucks, P., and D. F. Hales. 1996. Numerous transposed sequences of mitochondrial cytochrome oxidase I-II in aphids of the genus Sitobion (Hemiptera: Aphididae). Molecular biology and evolution **13**:510-524.

Swofford, D. L., and J. Sullivan. 2003. Phylogeny inference based on parsimony and other methods using PAUP*. The Phylogenetic Handbook: A Practical Approach to DNA and Protein Phylogeny, cáp **7**:160-206.

Tamura, K., and M. Nei. 1993. Estimation of the number of nucleotide substitutions in the control region of mitochondrial DNA in humans and chimpanzees. Molecular biology and evolution **10**:512-526.

Wilson, G. A., and B. Rannala. 2003. Bayesian inference of recent migration rates using multilocus genotypes. Genetics **163**:1177-1191.

Zelditch, M. L., D. L. Swiderski, and H. D. Sheets. 2012. Geometric morphometrics for biologists: a primer. Elsevier Academic Press, London, UK.
